# Prospects for combined use of oncolytic viruses and CAR T-cells

**DOI:** 10.1186/s40425-017-0294-6

**Published:** 2017-11-21

**Authors:** Adam Ajina, John Maher

**Affiliations:** 1 Department of Oncology, Royal Free London NHS Foundation Trust, London, UK; 2 King’s College London, CAR Mechanics Group, School of Cancer and Pharmaceutical Sciences, Guy’s Hospital Campus, Great Maze Pond, London, SE1 9RT UK; 3 Department of Clinical Immunology and Allergy, King’s College Hospital NHS Foundation Trust, London, UK; 4 Department of Immunology, Eastbourne Hospital, East Sussex, UK

**Keywords:** Oncolytic virus, Chimeric antigen receptor, CAR T-cell, Adoptive cell transfer, Combination strategies, Synergism, Solid tumours

## Abstract

With the approval of talimogene laherparepvec (T-VEC) for inoperable locally advanced or metastatic malignant melanoma in the USA and Europe, oncolytic virotherapy is now emerging as a viable therapeutic option for cancer patients. In parallel, following the favourable results of several clinical trials, adoptive cell transfer using chimeric antigen receptor (CAR)-redirected T-cells is anticipated to enter routine clinical practice for the management of chemotherapy-refractory B-cell malignancies. However, CAR T-cell therapy for patients with advanced solid tumours has proved far less successful. This Review draws upon recent advances in the design of novel oncolytic viruses and CAR T-cells and provides a comprehensive overview of the synergistic potential of combination oncolytic virotherapy with CAR T-cell adoptive cell transfer for the management of solid tumours, drawing particular attention to the methods by which recombinant oncolytic viruses may augment CAR T-cell trafficking into the tumour microenvironment, mitigate or reverse local immunosuppression and enhance CAR T-cell effector function and persistence.

## Background

This review focuses on the prospects for the synergistic combinatorial use of two distinct immunotherapeutic modalities – adoptive cell transfer (ACT) of chimeric antigen receptor (CAR)-expressing T-cells and oncolytic virotherapy. The latter has a long historical pedigree dating back to the 1950s, but has only very recently entered into routine clinical practice. This followed the approval of talimogene laherparepvec (or T-VEC), a recombinant granulocyte macrophage colony-stimulating factor (GM-CSF)-containing human herpes simplex type I virus (HSV-1), for inoperable locally advanced or metastatic malignant melanoma based upon compelling efficacy data from the phase III OPTiM trial [1]. T-VEC is currently being investigated in a number of early and late phase clinical trials in melanoma and other solid malignancies. These include its co-administration with immune checkpoint inhibitors targeted against programmed cell death protein 1 (PD-1; e.g. pembrolizumab or nivolumab), cytotoxic T lymphocyte antigen 4 (CTLA-4; e.g. ipilimumab) [2] or the combination of T-VEC with systemic chemotherapy [3] or radiotherapy [4, 5]. Several other oncolytic viruses (OVs) are also undergoing clinical evaluation (e.g. GL-ONC1 [6], vvDD [7]).

Clinical use of CAR T-cell therapy on the other hand has emerged within the last decade. This has followed on from the successful use of tumour infiltrating lymphocyte (TIL)-based ACT for patients with advanced melanoma, originally developed by Rosenberg and colleagues at the National Cancer Institute (NCI) in the late 1980s [8]. Autologous CAR T-cell therapy targeting the B-cell-specific cell surface protein CD19 have induced lasting and deep remissions in patients with refractory B-cell malignancies, such as acute lymphoblastic leukaemia (ALL) or chronic lymphocytic leukaemia (CLL) [9]. Several Phase II clinical trials investigating second generation anti-CD19 CARs have now reported and these agents are expected to enter routine clinical practice imminently. However, the development of effective CAR T-cell therapies for solid tumours has proved far less straightforward, owing to several critical obstacles pertaining to safety and potency. This review attempts to present opportunities to overcome these issues by highlighting potential for synergistic immunotherapy with oncolytic virotherapy.

## Oncolytic virotherapy: the story so far

The development of genetically engineered OVs came to the fore in the 1990s with the first clinical trials of recombinant adenoviruses, such as ONYX-015 [10]. For a long time, it was assumed that the dominant mechanism underpinning the anti-cancer effect of these agents stemmed from their oncolytic potential. It is now apparent, however, that the lysis of virally infected cancer cells plays a relatively indirect role in inducing tumour regression and long-term clinical benefit in most patients. Instead, it has become clear that clinical efficacy of OVs (such as T-VEC) is strongly dependent upon their ability to convert tumours into living “vaccine factories”. These provide immunological “danger signals” that include small molecules (e.g. uric acid [11] and adenosine triphosphate (ATP)), and protein mediators such as high-mobility group box 1 (HMGB1) [12] and type I interferon (IFN) signalling [13]. By this means, OV infection results in enhanced tumour-associated antigen presentation (due to neo-antigen spreading), improved T-cell and natural killer (NK) cell trafficking into the tumour microenvironment (TME) and enhanced effector function, leading to a “bystander effect” at local and distant sites of disease. Efficacy of oncolytic virotherapy is therefore dependent upon a complex interplay between functional innate and adaptive immune cells within the patient and more specifically within the TME itself. Many solid tumours present a significant barrier to this process whereby the TME is either non-permissive to entry of effector immune cells or exerts immunosuppressive effects on those cells that do manage to gain access [14]. The ability of recombinant OVs to modulate the TME is now being exploited further by rationally inserting transgenes to encode immunostimulatory cytokines, chemokines or co-stimulatory molecules into viral virulence genes, thus fulfilling a dual strategy of optimising tumour tropism and specificity. Oncolytic viruses are therefore highly attractive agents to use in combination with cellular therapies when targeting solid tumours.

A wide variety of OV vectors spanning numerous viral families have been identified and developed [13]. Pre-clinical and clinical studies are currently evaluating the potential of oncolytic adenoviruses, herpesviruses, poxviruses, picornaviruses (including coxsackievirus, polioviruses and Seneca Valley virus), paramyxoviruses (including measles viruses and Newcastle disease virus (NDV)), reoviruses, parvoviruses and rhabdoviruses (e.g. vesicular stomatitis virus (VSV)). The number of clinical trials evaluating OVs either alone or in combination with other therapies has expanded rapidly and these are summarised in detail in Table 1. Globally, two viruses, T-VEC and H101 have now achieved regulatory approval. H101 is a genetically modified oncolytic adenovirus that was approved in China in November 2005 for the treatment of nasopharyngeal carcinoma in combination with systemic chemotherapy [15]. The diversity of available OVs, each with their own hallmarks of tumour tropism and specificity, virulence and oncolytic potential allows for the nuanced and optimal selection of OVs for combined use with cellular therapies such as CAR T-cell therapy. Furthermore, many OVs have undergone extensive iterative laboratory-based study during the development of anti-viral vaccines over many decades. This provides reassurance with regards to safety and tolerability following administration in human subjects. Oncolytic strains of vaccinia virus – a large, complex, enveloped poxvirus – have the longest and most extensive history of administration in humans of any known virus due to their use in the eradication of smallpox during the middle of the last century [16].

**Table 1:**
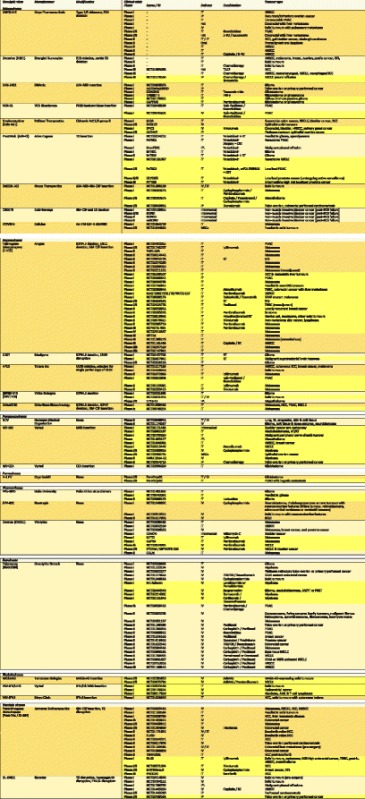
List of clinical trials evaluating OVs in solid tumours

Table updated and amended from [13]. Clinical trials highlighted in yellow are currently recruiting or not yet recruiting patients as of 28th August 2017 on clinicaltrials.gov. *AdMA3* Adenovirus with transgenic MAGE-A3 insertion, *AML* acute myeloid leukaemia, *AT/RT* atypical teratoid rhabdoid tumour, *BCG* Bacillus Calmette-Guérin, *CEA* carcinoembryonic antigen, *CNS* central nervous system, *CRT* chemoradiotherapy, *EGFR* epidermal growth factor receptor, *GM-CSF* granulocyte–macrophage colony-stimulating factor, *HAI* hepatic arterial infusion, *HCC* hepatocellular carcinoma, *hNIS* human sodium iodide symporter, *HNSCC* head and neck squamous cell carcinoma, *IFN-β* interferon beta, *IP* intraperitoneal, *IPL* intrapleural, *IT* intratumoural, *IV* intravenous, *MAGE-A3*, melanoma associated antigen 3, *MSC* mesenchymal stem cells, *MSI* microsatellite instability, *MV* measles virus, *NDV* Newcastle disease virus, *NSCLC* non-small-cell lung cancer, *PDAC* pancreatic ductal adenocarcinoma, *PNET* primitive neuroectodermal tumour, *RCC* renal cell carcinoma, *RGD* Arg-Gly-Asp motif, *RT* radiotherapy, *SCC* squamous cell carcinoma, *SCLC* small cell lung cancer, *STS* soft tissue sarcoma, *SVV*, Seneca Valley virus, *TACE* transarterial chemoembolization, *TK* thymidine kinase, *TNBC* triple negative breast cancer, *US11* unique short 11 glycoprotein, *VSV* vesicular stomatitis virus

As gene-manipulating technologies have moved to the forefront of bio-scientific research, great strides have been made in understanding and delineating the mechanisms of tumour tropism and specificity. Although this remains incompletely understood, it is recognised that many OVs are dependent upon cancer cells providing a nucleotide-rich environment and expressing relatively high levels of key molecules conducive to viral genomic replication, relative to normal tissue. Several mechanisms may underlie the tumour specificity of OVs. First, some OV achieve preferential viral entry into cancer cells by binding to cell surface molecules that are more highly expressed by certain tumours. This is illustrated by the ability of many OV strains of coxsackievirus to bind to intercellular adhesion molecule 1 (ICAM-1), which is a cell adhesion molecule that is over-expressed in many tumours [17]). Alternatively, OVs may exploit specific aberrant signalling pathways in cancer cells through one of many mechanisms. For example, vaccinia virus replication is favoured by heightened epidermal growth factor receptor (EGFR)-RAS signalling, as found in many solid tumours [18]. Similarly, overexpression of B-cell lymphoma (BCL) pro-survival proteins (such as BCL-xL) is targeted by NDV, which is able to continuously replicate and induce syncytium formation in apoptosis-resistant cells [19] while p53 deficient cancer cells are more susceptible to E1B deleted adenoviral strains [20]). The absence or impairment in cancer cells of type I IFN signalling renders these cells more susceptible to several OV strains [21]. Alternatively, some OV types exhibit preferential sequestration by the tumour microvasculature, as is seen with many vaccinia strains [22].

Many OVs such as adenoviruses and poxviruses have sufficiently large genomes to facilitate the insertion of foreign genes. The ability of recombinant OVs to modulate the TME is being exploited further by rationally inserting transgenes to encode immunostimulatory cytokines, chemokines or co-stimulatory molecules into viral virulence genes, thus fulfilling a second strategy aside from optimising tumour tropism and specificity [13]. Specifically, recombinant OVs can circumvent many of the tumour’s mechanisms of immune escape (e.g. by enhancing type I IFN signalling, upregulating major histocompatibility complex (MHC) class I expression on cancer cells [23], targeting enhanced transforming growth factor beta (TGF-β)/Wnt/β-catenin signalling and its negative impact upon antigen presentation [24] or by delivering inhibitors of active immunosuppressive pathways in the TME e.g. prostaglandin E2 (PGE2) [25] or adenosine A2a receptors (A2ARs). They may also deliver a therapeutic payload designed to enhance their oncolytic potential (e.g. apoptotic proteins such as apoptin [26] or death receptor ligands [27]).

Oncolytic viruses may be administered systemically or via intra-tumoural injection. This facilitates the broad application of OVs to specific combinatorial immunotherapeutic strategies. Both methodologies are associated with specific advantages and disadvantages. For example, the systemic delivery of OVs may be limited by the host’s defences. Viral particles may be sequestered by neutralising antibodies or by complement activation within the circulation; they may be filtered by the lungs, liver or spleen; and they may encounter physical barriers that limit their escape from the vascular compartment or prevent their entry into the TME [28]. Local instillation of OV into the tumour may bypass many of these barriers. However, due to their location many tumours are not immediately accessible to targeted OV delivery. They may be located deep within the body or in close proximity to critical structures. The systemic delivery of OVs also affords a method of targeting multiple metastatic deposits simultaneously. Several techniques have been explored in order to optimise the systemic delivery of OVs, such as by using cytokine preconditioning [29], complement inhibitors [30], immunomodulatory agents such as cyclophosphamide [31, 32], B-cell depleting agents such as rituximab or with plasmapheresis [33]. Transduced cytotoxic T-cells containing OV DNA have also been utilised as “Trojan horses” for ACT [34].

Currently there remain many stumbling blocks to the use of OVs as monotherapies in cancer patients. Aside from recent success seen in the field of malignant melanoma, only modest potency has been demonstrated in patients with other advanced solid tumours. One issue relates to the presence of pre-existing anti-viral antibodies in patients who have previously been vaccinated with similar vectors [28]. And in those who have not been vaccinated, the administration of an OV typically leads to the rapid development of immunity and viral clearance by neutralizing antibodies and complement. Other barriers to the efficient systemic delivery of OVs include aberrant tumour vasculature, mis-localisation and sequestration in non-target tissues and inadequate extravasation from the circulation [35]. Due to their putative immune-mediated mechanism of action, they require a relatively intact host innate and adaptive immune system. This is often compromised in cancer patients, whose relative immunodeficiency may also give rise to safety concerns due to unconstrained infection in non-target tissues [36]. A number of practical concerns have also curtailed their rapid development and the study of combination strategies may be impacted by the lack of OVs that have been approved for clinical practice. Technical and logistical challenges also exist that have limited the clinical evaluation of these agents outside of large academic centres.

## CAR T-cell immunotherapy: the story so far

Immunotherapy using CAR-engineered T-cells is undoubtedly one of the most innovative therapeutic strategies to have emerged among those that either co-opt or augment an individual’s capacity to mount an effective immune response against cancer. Chimeric antigen receptors are recombinant cell surface fusion molecules that couple the binding of a native tumour-specific or tumour-associated cell surface antigens (TSAs or TAAs) to the delivery of a bespoke T-cell-activating signal [37, 38]. CAR T-cells have proved efficacious in the management of patients with haematological malignancies and, in parallel with T-cell receptor (TCR)-gene modified antigen-specific T-cells, are currently being evaluated in patients with a variety of solid tumours. CAR T-cell therapy provides a number of advantages over TIL or TCR-engineered ACT. Firstly, CAR T-cells bypass the requirement for peptide processing, HLA expression and antigen presentation by cancer cells [37]. Given that the loss of MHC class I expression and the downregulation of proteasomal antigen processing are recognised as mechanisms of acquired resistance to cancer immunotherapy with immune checkpoint blockade [39] or standard ACT, this ability by CARs to circumvent the machinery of antigen presentation becomes particularly attractive. As a result, CAR T-cells can recognise antigen on any human leukocyte antigen (HLA) background, in contrast to TCRs which must be matched to a patient’s HLA haplotype [40]. Chimeric antigen receptors can also target non-protein TAAs such as carbohydrate or glycolipid structures [37]. Unlike TCRs however, they are limited to targeting cell surface rather than cytoplasmic or nuclear TAAs [40]. Whilst this does, to some extent, limit the potential repertoire of CAR targets, our increasing understanding of the “surfaceome” of both tumours and normal tissue is now providing a plethora of targets [41]. A number of these targets are currently being explored in early phase clinical trials and are summarised in Table 2. The ability to genetically engineer T-cells lends itself to limitless customisation and adaptation. In concert with the development of novel sensing and CAR-control technologies, this has the potential to inform the development of logic-gated stimulatory and inhibitory CAR circuits for the algorithmic targeting of tumours [42].

**Table 2:**
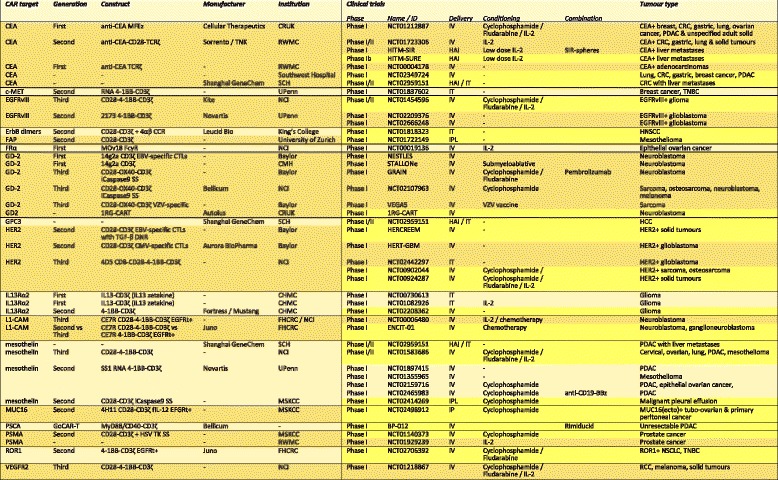
List of clinical trials evaluating CARs in solid tumours

Table updated and amended from [223]. Clinical trials highlighted in yellow are currently recruiting or not yet recruiting patients as of 28th August 2017 on clinicaltrials.gov. *CCR* chimeric costimulatory receptor, *CD19t* truncated CD19, *CEA* carcinoembryonic antigen, *CHMC* City of Hope Medical Centre, *CMV* cytomegalovirus, *c-MET* c-mesenchymal-epithelial transition, *CMH* Children’s Mercy Hospital, *CNS* central nervous system, *CRT* chemoradiotherapy, CRUK Cancer Research UK, *CTL* cytotoxic T-lymphocyte, *DNR* dominant negative receptor, *EBV* Epstein Barr virus, *EGFRt* truncated EGFR, *EGFRvIII* epidermal growth factor receptor variant III, *FAP* fibroblast activation protein, *FHCRC* Fred Hutchinson Cancer Research Centre, *fIL-12* feline interleukin-12, *FRα* folate receptor alpha, *GR* glucocorticoid receptor, *HAI* hepatic arterial infusion, *HCC* hepatocellular carcinoma, *HER2* human epidermal growth factor receptor 2, *HNSCC* head and neck squamous cell carcinoma, *HSV* herpes simplex virus, *HyTK* hygromycin phosphotransferase-HSV thymidine kinase, *IL-2* interleukin-2, *IL-13* interleukin-13, *IP* intraperitoneal, *IPL* intrapleural, *IT* intratumoural, *IV* intravenous, *MSKCC* Memorial Sloan-Kettering Cancer Centre, *NCI* National Cancer Institute, *NSCLC* non-small-cell lung cancer, *PDAC* pancreatic ductal adenocarcinoma, *RCC* renal cell carcinoma, *RWM* Roger Williams Medical Centre, *SCH* Shanghai Cancer Hospital, *SS* safety switch, *TCR* T-cell receptor, *TK* thymidine kinase, *TNBC* triple negative breast cancer, *UPenn* University of Pennsylvania

**Fig. 1:**
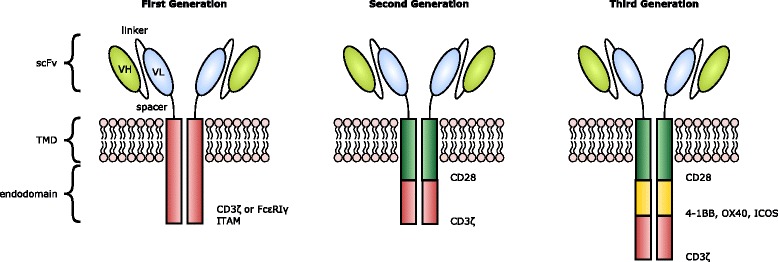
CAR design. CARs exist as dimers and consist of an ectodomain (typically comprising an scFv for target binding joined to an extracellular spacer e.g. IgG1 CH_2_CH_3_); a transmembrane domain (TMD); and a signalling endodomain. CAR design has evolved from first generation constructs linking the scFV to a CD3ζ or FcεRIγ-derived immunoreceptor tyrosine-based activation motif (ITAM) to second and third generation constructs, where the CARs endodomain contains one or two or more additional costimulatory molecules (such as CD28, 4-1BB, ICOS or OX40) [37]. Fourth generation CAR T-cells (not illustrated) termed TRUCKs are further modified with a constitutive or inducible expression cassette for a transgenic protein (such as IL-12), which is released by the CAR T-cells following receptor binding [101]

The design of CARs has undergone several iterative steps since their original description in 1989 by Eshhar and colleagues [43]. First generation CARs (termed “T-bodies”) incorporated an antigen-binding domain, such as an antibody-derived single chain variable fragment (scFv) or endogenous receptor ligand coupled to a CD8, CD4, CD25 or CD16 transmembrane domain and a CD3ζ or Fc receptor γ intracellular domain. Antigen engagement by the CAR induces the formation of an immune synapse with subsequent downstream signalling through a cascade of TCR-associated kinases. Ultimately, this leads to the transfer of cell-lysis inducing molecules (such as perforin and granzyme B) into the target cell, causing cytotoxicity as well as the secretion by the CAR T-cell of immunostimulatory cytokines that facilitate T-cell proliferation and activation in an autocrine and paracrine fashion [37]. However, first generation CAR T-cells failed to elicit a robust cytokine response with repeated antigen exposure and were susceptible to rapid onset of anergy [44]. Second generation CARs express both activating and co-stimulatory intracellular domains in cis and induce signalling that more closely mimics that of physiological TCR. In these receptors, a CD3ζ chain is fused to the cytoplasmic domain of a co-stimulatory receptor such as CD28, 4-1BB, OX40, ICOS or DAP10. Third generation CARs which are yet to demonstrate a clear improvement in efficacy incorporate three or more signalling domains e.g. CD28 and 4-1BB or CD28 and OX40, together with a source of signal 1 such as CD3ζ [45]. These CAR constructs are illustrated in Fig. 1.

Second generation CARs have demonstrated significant anti-cancer potency in pre-clinical and clinical models. On 30th August 2017 the United States Food and Drug Administration (FDA) approved the use of tisagenlecleucel (CTL019), a CD19-directed CAR T-cell product, for the treatment of patients up to 25 years of age with B-cell precursor ALL that is refractory or in second or later relapse. Approval was based on a number of early phase clinical trials including the pivotal single-arm, open-label, multicentre phase II ELIANA trial, which demonstrated that 83% of patients achieved complete remission (CR) or CR with incomplete blood count recovery within 3 months of treatment [46]. This agent, alongside other CAR products, has also been found to have considerable efficacy in other haematological malignancies such as chronic lymphocytic leukaemia (CLL) and lymphoma. Interim analysis presented at the International Conference on Malignant Lymphoma (ICML) meeting in Lugano, Switzerland, of the phase II JULIET trial evaluating tisagenlecleucel in adult patients with relapsed or refractory diffuse large B-cell lymphoma (DLBCL) revealed a 3 month overall response rate (ORR) of 45% with 37% achieving a complete response (CR) [47]. Results from the primary analysis of the phase II ZUMA-1 trial evaluating KTE-C19 (axicabtagene ciloleucel or axi-cel) in adult patients with relapsed or refractory DLBCL, primary mediastinal B cell lymphoma (PMBCL) or transformed follicular lymphoma were also presented at ICML. Treatment with axicabtagene ciloleucel was associated with an ORR of 82% with 39% in CR at a median follow-up of 8.7 months, a rate 7-fold higher compared to historical controls [48]. Furthermore, CAR T-cells with a central memory or stem-like phenotype can persist and remain efficacious for prolonged periods of time [49], as demonstrated in patients with durable remissions and B-cell aplasia following CD19-directed CAR T-cell therapy at a number of institutions such as the University of Pennsylvania [50, 51], Memorial Sloan-Kettering Cancer Centre [52, 53] and the National Cancer Institute [54, 55].

Whilst CAR T-cell immunotherapy has proved to be highly efficacious in patients with B-cell malignancies, this has not yet been reproduced in patients with solid tumours, which present several additional obstacles to success. These include: (i) a greater risk of unacceptable toxicity (due to potential for “on target off tumour” effects, caused by targeting TAAs expressed in multiple tissues) [56]; (ii) paucity of “dispensable antigens” expressed by solid tumours, unlike CD19^+^ B-cells [57]; (iii) poor trafficking of CAR T-cells into the TME [57] and (iv) impaired CAR T-cell effector function within the TME [58]. Another problem relates to the inherent heterogeneity of solid tumours, both temporally and spatially. Such variability is liable to result in incomplete tumour targeting by CARs and acquired resistance due to antigen loss [44]. In addition, the diversity of TAAs in solid tumours and distinct subtypes necessitates the design of suitably selective CARs for each disease entity.

The safety of CAR T-cell ACT remains a particular concern. Unfortunately, a number of clinical trials investigating CAR T-cell therapy have been marred by reports of fatalities due to severe cytokine release syndrome (CRS), macrophage activation syndrome (MAS) or neurotoxicity [59]. The latter, in particular, is poorly understood and has sometimes proved resistant to prevention or treatment with supportive strategies. There are also theoretical fears of insertional mutagenesis and the development of T-cell malignancy due to the use of integrating viral vectors, particularly those that exhibit powerful enhancer function [60].

The potential to cause “on target off tumour” toxicity is particularly pertinent to the design of CARs targeting TAAs expressed by solid tumours. This danger was highlighted by the death of a patient in a phase I first in-man clinical trial evaluating a third generation anti-HER2 CD28-4-1BB-CD3ζ CAR. In the aftermath of this case it was postulated that these potent HER2-directed CAR T-cells were able to recognise physiological low level HER2 protein expressed in normal lung tissue during their first pass passage through the pulmonary vasculature, leading to an inflammatory cytokine cascade, pulmonary toxicity and ultimately multi-organ failure [61]. More recently, numerous techniques have been explored to mitigate this risk and CAR products are currently in clinical development that incorporate inducible safety switches that can be triggered at will. Alongside the well-characterised herpes simplex virus-thymidine kinase (HSV-TK) / ganciclovir suicide gene system, CAR T-cells have been engineered to express an inducible caspase 9 gene (CaspaCIDe^®^) that induces apoptosis in the presence of rimiducid, a lipid-permeable tacrolimus analogue with homodimerizing activity [62]. An alternative strategy is to co-express a truncated cell surface protein, such as human epidermal growth factor (EGFR) that can be targeted by a pharmaceutical-grade monoclonal antibody, such as Cetuximab, leading to antibody-dependent cellular cytotoxicity [63].

In parallel, attempts have also been made to render CAR T-cell activation dependent upon the presence of an exogenously administered compound that is able to interact either with the CAR’s extracellular or intracellular domains. In the case of the GoCAR-T system, CAR T-cell proliferation, activity and cytotoxicity requires both TAA target binding as well as the presence of rimiducid, which facilitates homodimerization of an inducible chimeric MyD88/CD40 co-stimulatory domain [64]. The UniCAR modular system, on the other hand, incorporates a physiologically silent CAR that is activated in the presence of specific targeting modules. UniCAR T-cells may therefore be controlled more precisely in a time- and target-dependent fashion [65].

Finally, due to the requirement for use of intensive lymphodepleting conditioning regimens to facilitate CAR T-cell expansion, careful patient selection is required and currently the treatment remains suitable only for suitably fit patients without significant co-morbidity. Technical challenges and safety issues limit access to this approach outside of an academic centre experienced in the delivery of autologous haematopoietic transplantation. Furthermore, production of CAR T-cells is costly and time consuming due to the need to harvest, genetically engineer and expand autologous CAR T-cells ex vivo using cleanroom facilities. However, these issues may be addressed by centralised large-scale manufacturing, improved automation and a modular, integrated and scalable supply chain [66, 67]. Indeed batch manufacturing of Kite Pharma’s CD19-directed autologous CAR T-cell product axicabtagene ciloleucel can now be performed in just 6 days with a 2 week vein-to-vein turnaround time [68]. Following FDA approval of its CD19-directed CAR product tisagenlecleucel, Novartis have issued a price of US $475,000 per patient [69], which takes into account both the high manufacturing costs as well as the long-term benefits to young patients who would otherwise face costly bone marrow transplants, protracted hospital admissions and poor survival outcomes.

## Attributes of oncolytic virotherapy favourable to combined use with CAR T-cell therapy

The activation of pathogen or tumour-targeting CD8^+^ T-cells is dependent upon the presence of three classical signals: TCR engagement (signal 1), co-stimulation (signal 2) and an inflammatory stimulus (signal 3). Signal 3 is typically driven by cytokines such as interleukin (IL)-12 or type I IFNs [70]. Engagement of TAAs by second or third generation CARs provide engineered T-cells with signals 1 and 2. Whilst ex vivo activation of CAR T-cells by exposure to CD3/CD28 antibodies may recapitulate physiological signal 3 prior to administration, it remains unclear whether this signal remains present when T-cells enter the microenvironment of solid tumours and, if so, for what duration [71]. It is well recognised that type I IFNs can mediate anti-viral and anti-tumour responses by promoting viral eradication and limiting cellular proliferation, at least partly through a stimulatory effect on the host adaptive immune system. More specifically, type I IFNs support the proliferation, clonal expansion, effector function and/or memory formation of CD8^+^ T-cells [72]. Furthermore, IFNβ is also known to enhance cross-priming activity of dendritic cells (DCs), inhibit regulatory T-cell (Treg) activation and proliferation and disrupt the tumour microvasculature [73]. It is now recognised that OVs are capable of inducing an enhanced type I IFN signature in the TME. In concert with secondary enhanced DC and T-cell effector function and reduced regulatory T-cell (Treg) and myeloid-derived suppressor cell (MDSC)-induced immunosuppression, the immunophenotype of established tumours may thereby be converted from a so-called “cold” to a “hot” state that is permissive for CAR T-cell entry, expansion and efficacy [74].

Furthermore, studies of 4-1BB-containing second generation CARs have highlighted the potential importance of autocrine (and likely paracrine) feedback signalling via the secretion of IFNβ by the CAR T-cells themselves, reinforcing the importance of type I IFN signalling for effector T-cell function. It has been posited that the mechanism of type I IFN gene induction within CAR T-cells may occur via 4-1BB signalling through its activation of TNF receptor-associated factor 2 (TRAF2) [73]. CAR T-cells with enhanced 4-1BB signalling via 4-1BB-containing CARs or chimeric co-stimulatory receptors (CCRs) may therefore be particularly sensitive to type I IFN signalling within the TME and may prove particularly synergistic with OVs. Due to the complex modulation of effector T-cell function within the TME, signal 3 manipulation ex vivo is likely to have a profound and crucial influence on CAR T-cell behaviour in vivo [71].

**Fig. 2:**
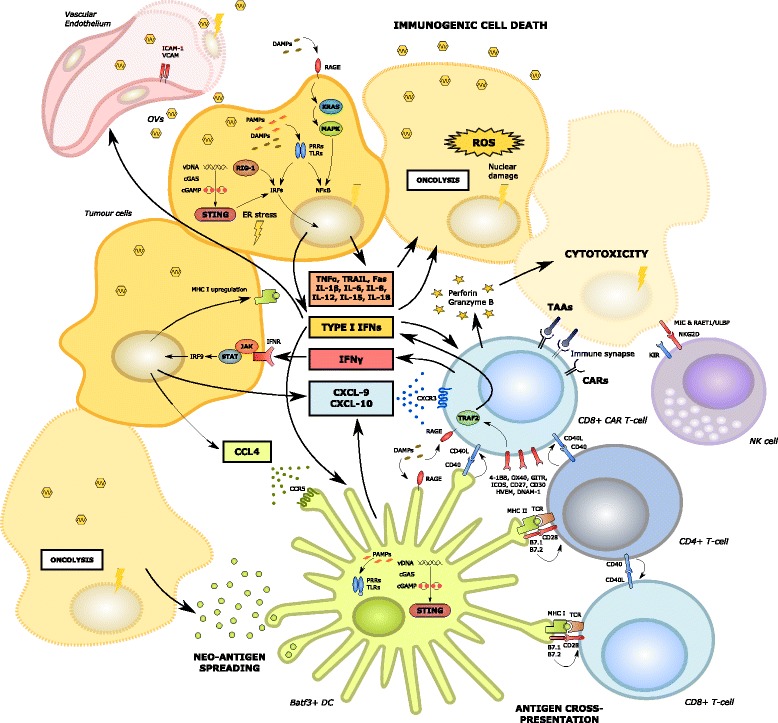
Oncolytic virus mediated enhanced anti-tumoral immunity, including enhanced CAR T-cell recruitment and effector function. Oncolytic viral infection of tumour cells induces immunogenic cell death (ICD) and a type I interferon response via release of PAMPs and DAMPs (such as HMGB1) acting on Toll-like receptors and RAGE. In addition, ER stress is induced by cGAS-cGAMP-STING pathway activation, ultimately leading to the phosphorylation of IRF and the transcription of type I interferons [13]. The local production of cytokines relevant to the activation of the innate immune system may be augmented by their delivery using recombinant oncolytic viral vectors. Activated DCs are recruited by the local production of CCL-4. In turn, DCs secrete CXCL-9 and 10 which attract CD8^+^ T-cells including CAR T-cells via CXCR3 [75]. Tumour cells with an intact interferon-sensing JAK-STAT pathway are also able to produce CXCL-9 and 10 and are induced to upregulate class I MHC [77]. Oncolysis induces neo-antigen spreading, enhanced DC function and antigen cross-presentation leading to the activation of anti-tumoural CD8^+^ and CD4^+^ T-cells within the TME [75]. The latter interact with CAR T-cells in a supportive manner, potentially via the expression of CD40 and other co-stimulatory molecules [141]. Oncolytic viral infection of local vascular endothelial cells may also induce the upregulation of adhesion molecules such as ICAM and VCAM

As described, OV infection and subsequent immunogenic cell death of cancer cells has been demonstrated to induce systemic innate and tumour-specific adaptive immune responses that impact upon T-cell trafficking and effector function within the TME (Fig. 2). Release of tumour neo-antigens and epitope spreading following OV-induced necrosis and pyroptosis of cancer cells leads to the recruitment of scavenging macrophages and Batf3^+^ dendritic cells, enhanced antigen presentation and subsequent activation of antigen-specific CD4^+^ and CD8^+^ T-cells. These T-cells are then able to traffic into tumour sites along chemokine gradients initiated by Batf3^+^ DCs in the TME [75]. Furthermore, OV infection can promote a permissive immunostimulatory milieu within the TME. The latter is facilitated by OV infection of cancer cells, leading to the release of viral pathogen associated molecular pattern signals (PAMPS). In turn, these trigger Toll-like receptor (TLR) activation, JAK-STAT signalling, the upregulation of viral clearance genes and local IFNα/β release [76]. This promotes the upregulation of MHC class I expression on cancer cells [77], which would be expected to be synergistic with ACT using TILs or TCR-engineered T-cells targeting specific tumour antigens such as the melanoma-associated antigen (MAGE-A) or New York oesophageal squamous cell carcinoma (NY-ESO) cancer-testis antigens. Nonetheless, CAR T-cells (which function independently of antigen presentation) would also benefit since their recruitment into the TME remains dependent upon chemokine signalling. Tumours with dysregulated MHC class I expression (e.g. due to acquired or intrinsic mutations in genes encoding β2-microglobulin or members of the JAK-STAT-IFNγ signalling pathway) [39] may be better targeted by CARs rather than engineered TCRs in conjunction with OVs.

Viral-induced oncolysis also induces local release into the extracellular environment of cellular danger associated molecular pattern signals (DAMPS) [78, 79] such as heat shock proteins, HMGB1, ATP, calreticulin and uric acid, as well as cytokines stimulatory of DCs and effector T-cells, including CAR T-cells, such as type I IFNs, tumour necrosis factor-alpha (TNFα), IFNγ, IL-12 and IL-15. HMGB1, in particular, can enhance T-cell activation and expansion within the TME via interaction with the T-cell Receptor for Advanced Glycation End Products (RAGE) [80, 81].

Furthermore, it has recently been demonstrated that the interaction of DNA OVs with the innate immune system’s cGAS-cGAMP-STING (Stimulator of Interferon Genes) cytosolic DNA sensing and signalling pathway may potentiate anti-cancer adaptive T-cell responses by inducing type I IFN gene transcription, DC activation and T-cell priming [82]. Type I IFN signalling is commonly disrupted in many different types of cancer and an important mechanism underlying this appears to be STING hypofunction, caused for example by inactivating mutations within tumours. This is emerging as a putative mechanism of intrinsic or acquired resistance to a number of immunotherapeutic modalities such as immune checkpoint blockade. Intriguingly however, STING-inactivated tumours have proven susceptible to DNA OV infection due to disruption of type I IFN signalling pathways [83]. In this setting, OV infection can induce sustained anti-cancer responses, implying that this may occur via mechanisms independent of intact type I IFN signalling or that OV infection may recapitulate IFN-regulated gene expression in these tumours. This suggests that combination OV and CAR T-cell therapy may be particularly efficacious in STING-inactivated and type I IFN disrupted tumours where OV infection may be more virulent [84] and the TME may otherwise prove too “cold” for ACT.

Enhancement of OV-induced type I IFN-mediated T-cell responses may be maximised by using a prime-boost approach with the sequential application of serologically distinct OVs [85]. In such a paradigm, it is posited that the adaptive immune response elicited by a second OV may be potentiated by the host immune response to the first OV, whilst simultaneously mitigating the potential immunodominance of specific viral antigens that may limit the development of robust anti-tumoural responses. Such an approach may provide even greater synergistic potential when combined with ACT and CAR T-cell therapy.

The capacity of OVs to facilitate the effects of TAA-specific ACT is illustrated in a chicken ovalbumin (OVA)-expressing murine melanoma model whereby intra-tumoural injection of an adenoviral OV could overcome resistance to the intra-peritoneal transfer of polyclonally activated OVA-specific CD8^+^ T-cells [86]. Adenoviral injection led to a local increase in pro-inflammatory cytokines, CD45^+^ leukocytes, CD8^+^ lymphocytes and F4/80^+^ macrophages as well as the induction of co-stimulatory signals on CD11c^+^ antigen presenting cells (APCs). As a result, T-cell activation occurred, which was accompanied by epitope spreading (evidenced by increases in CD8^+^ T-cells specific for the endogenous tumour antigens TRP-2 and gp100) and inhibition of tumour-induced peripheral tolerance.

Greater synergy may be achieved by combining adaptive anti-viral immunity with TAA-retargeted CAR T-cell therapy. To explore whether the former could enhance the efficacy and persistence of the latter, Epstein Barr virus (EBV)-specific T-cells were transduced with a first generation anti-GD2-CD3ζ CAR and compared with polyclonal redirected CAR T-cells in a phase I neuroblastoma study. Virus-specific CAR T-cells not only expanded more vigorously but were found to be more persistent in the short-term, being detectable by polymerase chain reaction (PCR) in patients 6 weeks following administration versus only 3 weeks in the polyclonal CAR T-cell treated cohort [87]. Building upon this approach, a further novel strategy may be to use OV vaccination prior to peripheral blood collection, T-cell selection and transduction with CARs targeting solid tumour antigens. Using this strategy researchers from Baylor College of Medicine stimulated peripheral blood mononuclear cells (PBMCs) obtained from patients who had received intratumoral JX-594, a recombinant vaccinia OV, with overlapping peptide libraries spanning the sequences of 6 vaccinia antigens. These were then transduced with a HER2-targeting CAR product and expanded. CAR T-cells specific for these viral antigens could secrete IFNγ in response to stimulation with vaccinia virus (VV) peptides in intracellular cytokine assays, suggesting that the efficacy of CAR T-cells primed to recognise OV antigens may be enhanced when used in conjunction with an OV vaccination schedule [88].

## Potential approaches to combine oncolytic virotherapy with CAR T-cell immunotherapy

**Fig. 3:**
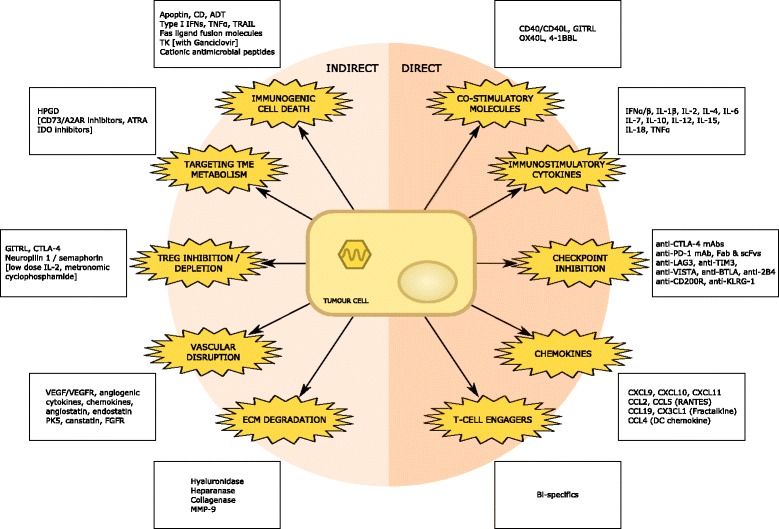
Recombinant oncolytic viruses with transgenes conferring direct and indirect synergism with CAR T-cell adoptive cell transfer. A large variety of oncolytic viruses have been engineered to express transgenes capable of augmenting responses to CAR T-cell therapies applied to solid tumours. These entities may either directly or indirectly enhance CAR T-cell efficacy by modulating their recruitment and entry within the TME, their activation and ability to kill tumour cells, their proliferative capacity, longevity and capacity to adopt a central memory phenotype. Many of these strategies attempt to target the immunosuppressive agents illustrated in Fig. 4 or augment many of the immunostimulatory characteristics highlighted in Fig. 2. Items enclosed in square brackets are pharmaceutical agents that may be synergistically combined with OVs & CARs

The flexibility of recombinant genetic engineering has led to a renaissance in the field of oncolytic virotherapy. A plethora of modified OVs are currently undergoing pre-clinical and clinical investigation, combining the favoured characteristics of impaired pathogenicity / virulence with enhanced oncolytic and/or immunostimulatory potential. Through precise editing of the viral genome, the oncotropic nature of these agents has been further enhanced. Similarly, such an approach may be used to develop experimental gene therapies that deliver a predefined therapeutic payload to the tumour, such as one or more pro-apoptotic proteins or immunogenic co-stimulatory surface molecules. Furthermore, the infection of tumours by oncolytic viruses has the potential to convert cancer cells into cytokine and chemokine factories, thereby converting the TME from an immunosuppressive to an immunostimulatory milieu that is permissive to T-cell entry and activation. This potential creates opportunities to develop exciting synergies with other immunotherapeutic modalities, including ACT and CAR T-cell therapy. In the paragraphs that follow, some examples of how engineered OV may be combined with CAR T-cells are considered. A summary of these strategies is illustrated in Fig. 3.

### Delivery of cytokines that support dendritic cells/ antigen-presenting cells

The canonical example of this type of engineered OV is T-VEC, whereby two copies of the human GM-CSF gene have been incorporated into the HSV-1 genome at the site of a deleted viral virulence gene (ICP34.5). Undeleted ICP34.5 can inhibit type I IFN activation and protein translation via blockade of the stress response protein kinase R (PKR) pathway. It is also able to inhibit IFNβ production by binding TANK-binding kinase 1 (TBK-1), preventing autocrine type I IFN signalling. ICP47, a second viral virulence gene that blocks antigen loading onto MHC class I molecules by binding to the transporter associated with antigen processing and presentation (TAP), is also deleted [76]. In the pre-clinical testing of T-VEC, regression of both injected and un-injected contralateral tumours was observed in immune competent mice, accompanied by a significant improvement in overall survival. These effects are mediated by improvements in antigen presentation and T-cell priming, attributes that would not be anticipated to be directly synergistic with CAR T-cell therapy, unless potentiation of endogenous tumour-reactive T-cells should prove pivotal. At a minimal level, it can be anticipated that CAR T-cell trafficking, activation and proliferation within the TME might all be enhanced due to the recruitment of DCs that modulate the cytokine milieu in favour of an adaptive immune response.

### Delivery of pro-T-cell cytokines

Cytokines have been the subject of considerable research due their pleiotropic anti-tumour effects. Following a greater recognition and understanding of the vitally important role that the host adaptive immune system plays in mediating OV responses, many researchers have focused on designing recombinant OVs capable of sculpting the cytokine milieu within the TME, thereby tipping the balance favourably from immune suppression to immune activation. By modulating the TME in this way, recombinant OVs render tumours more permissive to the entry of CAR T-cells, which may then benefit from a nurturing environment conducive to activation and expansion. However, care is required to ensure that the expression of cytokine transgenes does not result in a reduction in oncolytic activity and premature clearance of the virotherapy from the TME. Specific cytokine transgenes have been shown in pre-clinical models to enhance CAR T-cell activation and proliferation by promoting effector function, minimising the risk of exhaustion or hypofunction and modulating T-cell plasticity within the TME [89, 90]. Specifically, OVs have been designed to produce TNFα, IL-2, IL-4, IL-12, IL-15, IL-18 and type 1 IFNs [13, 91]. Several of these cytokines (particularly TNFα, TNF-related apoptosis-inducing ligand (TRAIL) and type I IFNs) may also induce direct cytotoxic effects on neighbouring uninfected cancer cells, depending upon their susceptibility [92]. Oncolytic HSV engineered to express a number of interleukin genes (including IL-12 and IL-4) demonstrated improved anti-tumour efficacy in murine glioblastoma models [93]. Similarly, oncolytic adenovirus co-expressing IL-12 and IL-18 was found to enrich tumour-specific immunity via the differentiation of T-cells [94]. Cytokine-encoding transgenes inserted into oncolytic vaccinia viruses include IL-1β, IL-2, IL-4, IL-6, IL-12 and IFNβ. Using a recombinant NDV vector, Bai et al. have demonstrated synergistic anti-cancer efficacy with transgenes encoding human IL-2 alongside TRAIL as well as others encoding IL-15 [95]. Arming OVs with type I IFNs has also been explored. When syngeneic LM2 lung tumours grown in the flanks of immune competent mice were injected with a VSV expressing IFNβ, researchers observed enhanced tumour regression, prolonged survival and cure in 30% of cases. Furthermore, VSV-IFNβ infection resulted in a decrease the numbers of tumour-infiltrating Tregs and an increase in CD8^+^ T-cells [96].

Interleukin-10 has been regarded for many years as a prototypic immunosuppressive cytokine that can inhibit T cell-mediated anti-viral and anti-tumoural responses [97]. However, more recently, pleiotropic effects have been elucidated, leading to a counter-argument to this view. Specifically, IL-10 has been shown to directly activate and expand tumour-resident CD8^+^ T-cells without promoting their de novo infiltration from secondary lymphoid organs [98]. A pegylated formulation of IL-10 is currently undergoing clinical evaluation and has demonstrated potent anti-tumour activity in patients with metastatic renal cell carcinoma [99]. A recombinant thymidine kinase (TK)-deleted Lister strain vaccinia OV armed with murine IL-10 was able to elicit tumour rejection in two murine pancreatic ductal adenocarcinoma (PDAC) models by prolonging the oncolytic potential of the OV and modulating innate and adaptive immune responses [100].

Several pre-clinical studies investigating so called “armoured” CARs or “trucks” have highlighted the potential of CAR T-cells being further engineered to secrete immunostimulatory cytokines capable of promoting enhanced efficacy via autocrine and paracrine effects [101]. Whereas, the systemic administration of IL-12 can lead to profound toxicity, expression of IL-12 by CAR-T cells was found to be safe in a syngeneic mouse model of CD19^+^ malignancies and intriguingly could obviate the requirement for lymphodepleting pre-conditioning, while rendering CAR-T cells resistant to Treg-mediated immunosuppression [102]. Similarly, arming CAR T-cells with IL-2, IL-7, IL-15 or IL-21 could enhance anti-lymphoma efficacy in an immune incompetent mouse model [103]. Such an approach may be replicated or enhanced by the local delivery of such cytokines to the TME by recombinant OVs.

Thus far, only a handful of pre-clinical studies have investigated the combination of cytokine-armed OVs with CAR T-cells. The combination of a mesothelin-directed second generation 4-1BB-containing CAR and an adenovirus construct containing either IL-2 or TNFα or both has been investigated in a preclinical mouse model of PDAC. Oncolytic virus infection was shown to enhance CAR T-cell efficacy in vivo and the IL-2 containing adenoviral vector was associated with enhanced T-cell numbers in mouse splenic tissue [104].

### Delivery of T-cell attracting chemokines

One of the most significant challenges faced in efforts to apply CAR T-cell therapy to solid tumours relates to impaired CAR T-cell trafficking into the TME. Bio-distribution studies following the systemic administration of CAR T-cells have demonstrated suboptimal trafficking into solid tumours due to mis-localisation / sequestration or by encountering physical or molecular barriers to entry [38]. Immediately following their systemic administration, CAR T-cells are prominently detected in the lungs owing to direct trafficking through the right ventricle and subsequent entrapment in the alveolar microvasculature [105, 106]. This can lead to significant toxicity in the presence of low levels of CAR-targeting TAA and presents a hurdle to subsequent CAR T-cell migration into the TME [61]. CAR T-cells that manage to enter tumour draining lymph nodes or the periphery of tumours themselves face the problem that chemokines produced by solid tumours and cell adhesion molecules expressed by endothelial cells in the TME vasculature do not favour T-cell infiltration [57].

One method that has been employed is the direct instillation of CAR T-cells into the TME by intra-tumoural or regional injection [107, 108]. Whilst this may be feasible for tumours that have spread in a purely local fashion, disseminated metastatic disease is unlikely to be effectively targeted in the absence of systemic CAR T-cell delivery due to the presence of physical and/or molecular barriers to effective T-cell migration. Furthermore, intra-tumoural injection can be both technically challenging and risky from a safety perspective in many solid tumours.

At the molecular level, it has been recognised that CAR T-cell trafficking is impacted by the inefficient and poorly coordinated expression of chemokines and cell adhesion molecules within the TME, including the luminal surface of tumour-associated lymphatic and vascular endothelium, and their target receptors on the CAR T-cells themselves. Chemokine ligand / receptor mismatching may stem in part from the imperfect replication of physiological TCR-mediated T-cell activation using second or third generation CARs. In recent years, studies in mice have shed light on the vital dual role played by Batf3^+^ dependent CD8α^+^ DCs in mediating anti-tumour adaptive immunity. It has become clear that murine CD8α^+^ (and potentially the human equivalent CD141^+^) DCs are actively recruited to the TME by tumour-associated chemokines (such as C-C motif chemokine ligand 4 (CCL-4)) and following their arrival are not only involved in the priming of CD8^+^ T-cells during antigen cross-presentation in the afferent limb of the anti-tumoural immune response but are also involved in the efferent limb by responding to danger signals via pattern recognition pathways (such as STING) by secreting type I IFNs and IFNγ-dependent chemokines (including C-X-C motif chemokine ligand 9 (CXCL-9) and CXCL-10) that attract primed effector T-cells into the TME via specific chemokine receptors, most notably CXCR3 [75]. This concept is particularly relevant for CAR T-cell therapy which is designed to bypass the afferent limb altogether. Using Batf3^+^ knock-out mice, Gajewski and colleagues showed that type I IFN-mediated signalling in Batf3^+^ dependent CD8α^+^ DCs was critical for the rejection of immunogenic tumours [109]. The exogenous delivery of the DC-derived cytokine IL-12 can recapitulate anti-tumour immunity associated with enhanced IFNγ signalling within the TME [110]. The implication is that in addition to the CD28 co-stimulatory signal normally required for T-cell priming, CAR T-cells are likely to require the presence of an inflamed TME with active Batf3^+^ dependent CD8α^+^ DCs contributing to type I IFNs and IFNγ-mediated CXCL-9/10 secretion. In the absence of this mechanism, (e.g. in tumours with an activated Wnt/β-catenin pathway leading to transcriptional repression of CCL-4 [111]), impaired trafficking and function of Batf3^+^ dependent CD8α^+^ DCs and a non-inflamed tumour immunophenotype are likely results. For these reasons, CAR T-cells are likely to benefit from the synergistic combination with OVs which recapitulate the required type I IFN signature, as well as deliver the required chemokine ligands by genetic recombination.

The importance of the C-X-C motif chemokine ligands CXCL-9 and CXCL-10 in mediating adaptive anti-tumour immunity is reinforced by their repeated identification in RNA-Sequencing transcriptome analysis of tumours with an “inflamed” immunophenotype predictive of response to immune checkpoint blockade [112, 113]. Indeed, a number of clinical studies have highlighted the prognostic and predictive value of both CXCL-9 and CXCL-10 in patients receiving treatment for colorectal, breast and high grade serous ovarian cancer [114–116]. The natural ability of OVs to enhance levels of CXCL-9 and CXCL-10 locally within the TME has been exploited in pre-clinical studies investigating adoptive T-cell transfer where the presence of these chemokines facilitated T-cell migration and persistence within the TME, leading to a significantly enhanced therapeutic effect [117]. Additionally, a number of researchers have sought to arm OVs with these chemokine ligands in an effort to exploit their ability to modulate the TME and reverse inefficient trafficking and local expansion of cytotoxic T-cells (as well as other immune cells such as NK cells, macrophages & DCs) [118, 119].

Intra-tumoural injection of a tumour-selective oncolytic double-deleted vaccinia virus (vvDD) armed with the chemokine CXCL-11 was found to enhance the recruitment of tumour-specific CD8^+^ effector T-cells into the TME in a murine AB12 mesothelioma model, leading to locally elevated levels of granzyme B and reduced expression of several suppressive molecules including TGF-β, cyclooxygenase 2 (COX2) and CCL-22. It was also associated with an induction of systemic anti-tumour immunity with an increase in tumour-specific IFNγ-producing CD8^+^ T-cells in the spleen and other lymphoid organs and ultimately was associated with a survival benefit in treated mice [118].

A vaccinia strain of OV (vvDD) designed to constitutively express the chemokine CCL-5 (regulated on activation, normal T cell expressed and secreted (RANTES)) was found to increase immune cell infiltration in a mouse colorectal tumour model leading to enhanced therapeutic effects. Interestingly, vvCCL-5 demonstrated a heightened capacity to persist for extended periods in tumours in vivo [120]. Whereas in the absence of OV infection enhanced tumour production of CCL-5 resulted in Treg recruitment and progression, vvCCL-5 infection led to CD4^+^ effector T-cell infiltration and a Th2 skewed immune response. Demonstrating how combination strategies with OVs may further modulate the TME and enhance anti-tumoural adaptive responses, greater efficacy was achieved when vvCCL-5 was combined with DC1 vaccination – an approach known to induce high levels of activated cytotoxic (Tc1) T-cells expressing CCL-5 receptors – leading to further enhanced immune cell infiltration and Th1-skewing.

The systemic or intraperitoneal administration of a recombinant vaccinia OV (vvDD) expressing the chemokine CCL-19 was found to result in enhanced anti-tumour effects in syngeneic mouse tumour models. The lymph node targeting receptor for CCL-19, CCR7, is expressed in a relatively more restricted set of immune cells (including mature DCs, T-cells and cytokine induced killer (CIK) cells) compared to the CCL receptors CCR1, CCR3, and CCR5 [121]. Expression of CCL-19 did not curtail oncolytic activity and vvCCL-19 was cleared rapidly and selectively from normal tissues suggestive of a potentially enhanced safety profile. The researchers posited that the therapeutic activity of vvCCL-19 could be further improved through combination with ACT of immune cells overexpressing CCR7, and such an approach lends itself well to synergism with engineered CAR T-cell therapy.

The use of chemokines and their receptors may be a double-edged sword in cancer immunotherapy. Concerns due to the potential pro-tumour effects of the C-X-C chemokine ligands in particular have been highlighted in a number of cancers [122]. Whilst many CXCL-9-poor tumours have a demonstrably poorer prognosis (presumably due to reduced adaptive anti-cancer immunity) others appear to derive growth and invasion signals from CXCL-9 (e.g. via over-expression of the CXCR3 receptor [123]). For example, pre-treatment serum CXCL-9 levels in patients with nasopharyngeal carcinoma (NPC) were found not only to be significantly higher in those with higher stage disease but predicted for poorer prognosis in terms of overall survival and disease-free survival [124].

This disparity may be due in part to the diversity of chemokine targets and functional differences exhibited by individual chemokine receptor isoforms. For example, studies have demonstrated two main sub-types of the CXCR3 receptor: CXCR3-A and CXCR3-B, which may be expressed in a differential fashion on the surface of immune cells and tumour cells. A third sub-type, CXCR3-alt, is a poorly understood variant that is generated by the alternative splicing of mRNA encoding CXCR3-A and fails to signal in response to CXCL-9 or CXCL-10 [125]. When expressed on tumour cells CXCR3-A is pathogenic, interacting with CXCL-9 to promote tumour migration and invasion via the activation of phosphatidylinostol 3-kinase (PI3K) and mitogen activated protein kinase (MAPK) signalling pathways. By contrast, CXCR3-B is anti-angiogenic in this context, interacting with CXCL-9 to depress endothelial cell proliferation and tumour angiogenesis. Expression of CXCR3-A on T-cells fosters chemotaxis and is immunogenic. Furthermore, due to the overlapping targets of chemokine ligands and their pleiotropic effects on a variety of immune cells, there may be concerns that the overexpression of chemokines by OVs may have unintended consequences in specific tumour models. For example, in a colorectal cancer model neuroendocrine differentiation may induce the infiltration of tumour-associated macrophages (TAMs) attracted by CXCL-10 and CXCL-11 leading to enhanced tumour cell proliferation and invasion [126]. CXCL-9 may also negatively impact on the TME by attracting Tregs and inducing CXCR3^+^ B-cells to polarise immunoregulatory (M2b) TAMs [127]. The chemokine fractalkine (CX3CL1) has also been implicated in the invasive and metastatic potential of several tumours including lung, ovarian and prostate cancer. In ovarian cancer, high expression of the fractalkine receptor CX3CR1 correlated with significantly shorter survival in post-menopausal patients with advanced disease [128].

Oncolytic viruses have therefore been designed to target the pro-tumour effects of specific chemokine ligand / receptor interactions that are believed to play a role in tumour cell extravasation and metastasis. For example, the systemic delivery of a vaccinia OV armed with a CXCR4 antagonist to mice with orthotopic mammary tumours disrupted the interaction between tumour cell CXCR4 and CXCL-12 present in the stromal microenvironment. This lead to tumour growth retardation, a reduction of spontaneous metastasis, the destruction of tumour associated vasculature and increased overall tumour-free survival [129].

### Delivery of pro-T-cell cytokines & chemokines in combination

Nishio and colleagues have demonstrated that Ad5Δ24 (an oncolytic adenovirus armed with the chemokine CCL-5/RANTES and the cytokine IL-15) could be used synergistically with 3rd generation human CAR T-cells (specific for GD2 and containing the CD28 and OX40 intracellular signalling domains) in a neuroblastoma-bearing immune compromised NOD SCID γc^null^ (NSG) mouse model. Specifically, OV infection directly accelerated the caspase-dependent death pathways within tumour cells exposed to CAR-T cells and the intra-tumoural release of both CCL-5 and IL-15 attracted CAR-T cells and promoted their survival within the TME, thus increasing the overall survival of the mice studied [130, 131]. Naturally, by using an immunodeficient mouse model, any potential interplay between the OV, CAR T-cells and inhibitory immune cells such as Tregs, TAMs and MDSCs cannot be properly assessed. This will require additional study using either murine CAR T-cells in an immune competent mouse model or by using appropriate humanised mouse models [132].

### Delivery of immune co-stimulatory molecules

Oncolytic viruses have also been armed with co-stimulatory molecules to enhance the local activation and expansion of effector immune cells within the TME. In studies without combination ACT, arming adenovirus and vaccinia vectors with TNF receptor superfamily ligands including OX40L, 4-1BBL, and GITRL led to enhanced anti-tumour effects [133–135]. In addition, OVs have been armed with transgenes encoding membrane bound or soluble CD40 ligand (CD40L), thereby enhancing local CD40 activation within the TME [136, 137]. Due to the variable expression of CD40 on a wide variety of immune cells, including CD4^+^ helper T-cells, macrophages and B-cells, activation of this pathway mediates anti-tumour immunity in a pleiotropic manner [138]. For example, a novel oncolytic adenovirus armed with trimerized membrane-bound extracellular CD40L led to the activation of myeloid and endothelial cells, supporting T-cell expansion and migration into the TME. The researchers hypothesised that CD40L-mediated gene therapy may prove to be particularly promising for the targeting of tumours with high levels of M2 macrophages, such as PDAC [139]. Furthermore, the enhanced natural expression of CD40L on CD8^+^ effector memory T-cells and our emerging understanding of the role of CD40/CD40L interactions (particularly between CD4^+^ and CD8^+^ T-cells) in supporting an effector memory phenotype [140, 141] suggests that the activation of this pathway may be particularly synergistic with ACT including CAR T-cell therapy. This approach has also been explored in the ACT space by arming CAR T-cells with constitutively expressed CD40L [142]. CD40L-modified T-cells demonstrated increased proliferation and secretion of pro-inflammatory Th1 cytokines and an ability to induce monocyte-derived DC maturation and secretion of IL-12.

Oncolytic viruses have also been armed with B7-1 (CD80), a co-stimulatory molecule overexpressed by mature DCs at the time of antigen cross-presentation mediated effector T-cell activation. Specifically, a vaccinia virus engineered to either express B7-1 alone or in combination with the cell adhesion molecules ICAM-1 and lymphocyte function-associated antigen 3 (LFA-3) (the triplet combination termed TRICOM) can provide an optimised form of co-stimulation required by anti-tumour CD8^+^ T-cells and has demonstrated efficacy and tolerability in several early-phase clinical trials in patients with malignant melanoma [143]. In these studies, adverse events were mild, consisting of flu-like symptoms and local injection site reactions. Interestingly, several patients subsequently developed autoimmune vitiligo.

### Delivery of molecules targeting immune checkpoints

The efficacy of TIL-based and other forms of ACT may be subject to numerous inhibitory immune-checkpoint molecules able to exert their effects at various stages of effector T-cell activation and expansion. These include CTLA-4, PD-1, B7-H family members and Fas ligand [144]. Numerous additional T-cell suppressive cell surface molecules are currently being characterised and the complexity of immunosuppressive signalling within the TME is further apparent when one considers the dynamic temporospatial expression of these molecules. Many of the effects seen in TIL therapy are applicable to CAR T-cell therapy. For example, both TCR-engineered and CAR-T cells routinely express PD-1 and are susceptible to PD-L1/PD-L2-mediated suppression [145, 146]. Furthermore, CAR T-cell hypofunction in the TME has been shown to be associated with the up-regulation of intrinsic T-cell inhibitory enzymes, including diacylglycerol kinase (DGK) and SHP-1, and the expression of cell-surface inhibitory receptors including PD-1, LAG3, TIM3 and 2B4 [147].

Whilst it would be anticipated that CAR T-cell therapy could be synergistically enhanced if used in combination with licensed immune checkpoint inhibitors (such as mAbs targeting PD-1, PD-L1 or CTLA-4), recombinant OVs armed with these agents may replicate this function in a more targeted, safe and nuanced manner as well as providing a plethora of other pro-inflammatory signals conducive to innate and adaptive anti-tumour responses. In keeping with this, a recombinant adenoviral OV has been armed with a full length IgG2 mAb targeting human CTLA-4 [148]. Murine PD-1 has been successfully targeted with a Western Reserve (WR) oncolytic vaccinia virus armed with either a full-length hamster monoclonal IgG antibody, a fragment antigen-binding (Fab) fragment or a single-chain variable fragment (scFv) [149]. Intra-tumoural injection of these OVs induced a very significant infiltration of immune cells into the TME of melanomas and fibrosarcomas in immune competent mice. In the latter, OVs armed with whole antibody or scFv mediated superior anti-tumour effects compared to the unarmed virus. Furthermore, due to their ability to induce epitope spreading, OVs armed with anti-PD-1 molecules may also actively limit acquired resistance to immune checkpoint blockade by broadening neoantigen-directed T-cell responses.

In a similar fashion, OVs armed with soluble PD-1 traps or secreted antibodies/scFvs targeting other cell surface immune checkpoints such as TIM3, LAG3, VISTA, BTLA, CD200R, KLRG-1 and 2B4 would be expected to function synergistically with CAR T-cell therapy. Several similar strategies are also being employed in the design of novel CAR T-cells that have been engineered, for example, to secrete PD-L1 antibodies [150] or co-express a cell surface PD-1 dominant negative receptor [151]. One may also envisage arming OVs with intrabodies (intracellular antibodies), inhibitory short hairpin RNAs (shRNAs) or ribozyme switches targeting cancer cell surface co-inhibitory molecules such as PD-L1, B7-H, CD47 (inhibiting phagocytosis by macrophages) or other inhibitory ligands.

### Delivery of molecules targeting immunosuppressive cells

**Fig. 4:**
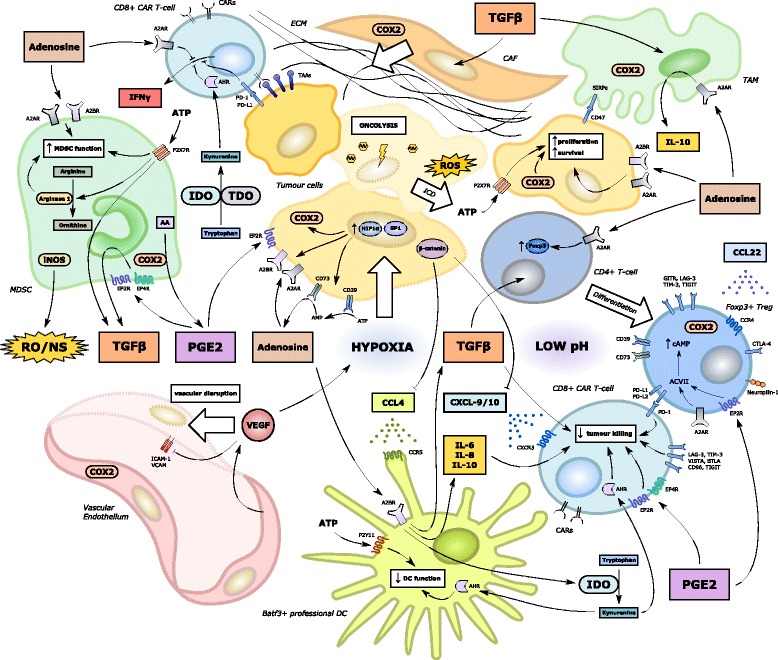
Immunosuppressive influences on CAR-T cell effector function within the tumour microenvironment. A large number of molecular and cellular players have been implicated in the development of an immunosuppressive milieu non-conducive to anti-tumoural T-cell recruitment, trafficking and effector function. Successful implementation of CAR T-cell therapy for solid tumours will necessitate the targeting of many of these players. Key environmental factors include: intra-tumoural hypoxia (exacerbated by VEGF); low pH (in part due to tumour cell lactic acid production); deficiencies of critical or semi-critical amino acids (e.g. tryptophan via IDO1/TDO or arginine via arginase 1 respectively); high levels of ATP and adenosine; increased COX activity and production of PGE2; high levels of immunosuppressive cytokines such as TGFβ and IL-10; upregulation of immune checkpoints on tumour cells and immune cells (particularly PD-L1, LAG-3 and TIM-3); the presence of a relatively impenetrable ECM; and high levels of reactive oxygen and nitrogen species [58]. Many of these factors either directly limit CAR T-cell function or augment the differentiation, recruitment, proliferation and immunosuppressive function of local immune cells within the TME such as Tregs, MDSCs, TAMs and CAFs [14]. DCs are also rendered dysfunctional and whilst CARs can target TAAs or TSAs independent of DC cross-priming, CAR T-cell trafficking and effector function are likely to be negatively impacted by DC dysfunction and loss of CXCL-9/10 chemokine signalling particularly [75]. In addition, high levels of VEGF and hypoxia may limit CAR T-cell entry by causing local disruption of the vascular endothelium and the downregulation of cell surface adhesion molecules [223] to aid CAR T-cell rolling and intra-tumoural migration

A very significant obstacle to effective CAR T-cell therapy in solid malignancy relates to the immunosuppressive signalling mediated by a variety of immune cells within the TME. The most commonly encountered players in this milieu include Tregs, TAMs, MDSCs, fibroblasts and endothelial cells [57]. This challenging environment is illustrated in Fig. 4, where several immunosuppressive environmental factors sustain effector T-cell exclusion and impaired function within the TME. A great deal of work has been conducted exploring how OVs can negate TME immunosuppression and reverse signalling that is non-permissive to CD8^+^ T-cell expansion and effector function. These strategies would be expected to combine synergistically with ACT and CAR T-cell therapy.

Transforming Growth Factor-β and Tregs are major contributors to the formation of immunosuppressive networks within the TME. The former suppresses the activation, maturation, and differentiation of immune cells such as effector T-cells, NK cells, and DCs [23] and is also a vital enabler of the maintenance and proliferation of Tregs, which further enhance local immunosuppression by autocrine secretion of TGF-β and IL-10 [152]. Decorin is a prototypic member of the small leucine-rich proteoglycan family and blocks the interaction between TGF-β and its receptor. In a murine orthotopic mammary tumour model, infection with a recombinant adenoviral OV armed with IL-12 and decorin limited the number of Tregs in draining lymph nodes and tumour tissues while promoting enhanced intra-tumoural infiltration of CD8^+^ T-cells [153].

The complexity of cellular interaction within the TME may reveal unforeseen factors with regards to OV infection. Tumour secretion of TGF-β is known to enhance the transition of normal fibroblasts to a cancer-associated fibroblast (CAF) phenotype, via epigenetic regulation [154]. In turn, CAFs can induce epithelial-mesenchymal transition (EMT) of tumour cells through paracrine TGF-β signalling [155]. Cancer-associated fibroblasts have also been found to be more susceptible to infection by a variety of OVs and may thus act as a decoy reservoir of infection [156]. However, the infection of CAFs was found to lead to enhanced secretion of fibroblast growth factor 2 (FGF2) leading in turn to impaired evasion of tumour cells to OV infection [157]. Furthermore, a Maraba MG1 virus encoding human FGF2 was found to have enhanced oncolytic potential in tumours but failed to replicate in or kill normal cells.

Glucocorticoid-induced TNFR-related protein (GITR) is a cell surface receptor that is constitutively expressed at high levels on Foxp3^+^ Tregs and at low levels on naïve and memory T-cells [158]. Upregulation of GITR occurs rapidly in effector T-cells via canonical NFκB signalling following activation. Binding of cognate ligand (GITRL) promotes proliferation, cytokine production [159], resistance to Treg suppression [160] and inhibition of Treg suppressive function [161]. Using in vivo experimental models, the administration of a GITR agonist antibody is associated with reduction of intra-tumoural Treg accumulation and potentiation of anti-tumour CD8^+^ effector T cell function, leading to enhanced anti-tumour effects [162]. It is to be expected that recombinant OVs armed with membrane-bound or soluble GITRL may enhance effector T-cell function by abrogating Treg-mediated immunosuppression. In support of this, intra-tumoural injection of melanomas with an adenovirus expressing either soluble or full length GITRL led to increased local CD4^+^ and CD8^+^ T-cell infiltration compared to controls [135]. Furthermore, tumours receiving soluble rather than full length GITRL exhibited greater growth retardation.

Conditioning regimens have also been utilised to deplete Tregs prior to OV administration and overlap with strategies used in ACT and CAR T-cell therapy. For example, utilisation of a non-toxic dose of IL-2 resulted in mild vascular leak syndrome which, accentuated by the depletion of Tregs, facilitated the localization of a systemically delivered vesicular stomatitis virus (VSV) OV in established tumours in immune-competent mice [163].

Use of the monoclonal antibody (mAb) ipilimumab to target the CTLA-4 pathway has been a standard of care in patients with advanced malignant melanoma for a number of years. Ipilimumab is known to disrupt effector T-cell expansion by binding to cell surface CTLA-4, which is upregulated on the T-cell surface following antigen cross-presentation by DCs. Moreover, a number of studies have highlighted the importance of Treg inhibition in contributing to ipilimumab’s clinical efficacy [164]. Regulatory T-cells constitutively express high levels of CTLA-4 which can bind directly to B7 cell surface molecules found on both DCs and activated T-cells, inducing immunosuppressive circuits and tolerance. The induction of potent and non-specific autoimmune toxicity following systemic delivery of ipilimumab has led to safety concerns. This is particularly the case when ipilimumab is combined with anti-PD-1 immune checkpoint inhibitors and may in part be the result of systemic rather than local inhibition of Tregs. An oncolytic adenovirus (Ad5/3-Δ24aCTLA4) has been armed with a complete human IgG2 subtype mAb specific for CTLA-4 and was tested in vitro, in vivo and in PBMCs of normal donors and patients with advanced solid tumours. Infection with this virus induced high local levels of anti-CTLA-4 mAb in the TME compared to blood [148]. However, the impact on host Tregs could not be assessed in a murine model when targeting human CTLA-4.

Finally, OVs may be armed with secreted inhibitors of Treg-attracting chemokine ligands (such as CCL-1, CCL-17 and CCL-22 [165]), Treg chemokine receptors (such as CCR4 and CCR8), or Treg-derived suppressive cytokines other than TGF-β (such as IL-10 and IL-35). Recombinant OV-mediated disruption of the neuropilin 1 / semaphorin signalling pathway may also constitute a novel strategy for inducing Treg depletion and reduced suppressive function within the TME [166].

Tumours rich in MDSCs are poorly accessible to effector T-cells and generally respond poorly to immunotherapy with checkpoint blockade. The immunophenotype of such tumours have been characterised using gene signatures and they are often termed “mesenchymal” e.g. consensus molecular subtype 4 (CMS4) in colorectal cancer [167]. Few strategies are currently available in the clinic to target such tumours. However, an oncolytic vaccinia virus expressing the PGE2-inactivating enzyme 15-prostaglandin dehydrogenase (HPGD) was found to significantly reduce both PGE2 and the number of infiltrating granulocyte-like (G)-MDSCs within the TME via a reduction in local CXCL-12 levels [25]. The importance of targeting mechanisms of immune suppression as well as enhancing immune activation is illustrated in the same model where immune-enhanced vaccinia strains (including WR.TK^-^mGM-CSF) were unable to elicit effective anti-tumour adaptive immunity in tumours with high baseline levels of G-MDSCs.

Immunotherapy using a GD2-redirected 3rd generation CD28 OX40 CD3ζ CAR in a mouse xenograft neuroblastoma model was found to be augmented using all-trans retinoic acid (ATRA) which was able to eradicate monocytic MDSCs and diminish the suppressive capacity of G-MDSCs which otherwise limited CAR T-cell activity in the TME [168]. Such an effect is likely to be mediated by ATRA’s known ability to impair the proliferation of CD34^+^ precursor cells and curtail MDSC myelopoiesis [169]. While ATRA has been explored as an adjuvant therapy in conjunction with a suicide gene carrying adenoviral vector (Ad-TK/GCV) [170] its potential to enhance the synergistic combination of OVs with cellular therapies remains to be explored. Likewise, OVs armed with enzymes and proteins required for ATRA synthesis such as RDH10, RALDH2 and CRABP2 remain to be developed.

### Delivery of molecules enhancing T-cell and tumour cell engagement

Recombinant OVs have been designed to enhance the engagement of effector T-cells with tumour cells by being armed with bispecific adapter proteins [171, 172]. Alternatively, these molecules have been utilised as accessory pharmacotherapies to enhance the adaptive immune response generated by oncolytic virotherapy [173]. These molecules termed bispecific T-cell engagers (BiTEs) have already entered into clinical practice following the approval of Blinatumomab, a BiTE linking anti-CD3 with anti-CD19, for the treatment of Philadelphia chromosome-negative relapsed or refractory B-cell precursor ALL [174]. Haas and colleagues have developed a recombinant bispecific single-chain antibody with one arm specific for NDV-expressing hemagglutinin-neuraminidase (HN) molecules and the other arm specific for the CD3 or the CD28 antigen on human T-cells [173]. The cross-linking of NDV-infected tumour cells with effector T-cells led to enhanced polyclonal T-cell-mediated cytotoxicity at nanomolar concentrations in vitro. Anti-tumour efficacy was further enhanced when PBMCs or purified T-cells were co-incubated with this agent for 3 days and then serially transferred to new tumour cell monolayers.

Wang et al. have developed a novel T-cell engager armed vaccinia virus (TEA-VV) that, following the infection of tumour cells, results in the secretion of potent bispecific antibodies binding both T-cell-associated CD3 and the tumour-associated cell surface antigen EphA2 [172]. The resulting EphA2-TEA-VV displayed significantly enhanced efficacy by inducing the bystander killing of tumour cells not yet infected with virus. Furthermore, the researchers investigated a potentially synergistic paradigm with CAR T-cell therapy by demonstrating that EphA2-TEA-VV could replicate in HER2-redirected CAR-T cells. Furthermore, the presence of Eph2-TEA-VV in co-culture assays with CAR T-cells could strongly enhance the killing of HER2^+^EphA2^+^ A549 tumour cells. Aside from the independent anti-tumour effects of HER2-redirected CAR-T cells recognising and lysing HER2 positive tumour cells and the oncolytic potential of the vaccinia virus, the researchers highlighted the synergistic potential of the locally secreted bi-specific antibody expressed by EphA2-TEA-VV directing T-cells to recognise and kill EphA2-expressing tumour cells, overcoming tumour heterogeneity. Such a strategy may circumvent the need to co-express CARs targeting more than one TAA and may exploit the multifarious mechanisms by which OV-infection can render the TME more permissive to CAR T-cell entry and activation.

In a similar vein, one may envision the design of a recombinant OV capable of inducing the local secretion of small protein-linked Fabs or scFvs targeting specific TAAs e.g. HER2 or PSMA. A cognate CAR may then be designed to target a homologous protein moiety within the Fab in a similar fashion to the “uniCAR” system [65]. These Fabs could then act as locally delivered bridging molecules for “off the shelf” CAR T-cell therapy.

### Delivery of molecules targeting immunosuppressive metabolic pathways within the tumour microenvironment

Both OV infectivity and CAR T-cell function may be compromised by a hostile metabolic milieu in the TME (illustrated in Fig. 4). Negative factors that impact on both include tumour hypoxia, oxidative stress, tryptophan depletion due to high IFNγ-induced indoleamine 2,3-dioxygenase 1 (IDO1) levels and low pH [58]. Factors impacting predominantly upon CAR T-cell efficacy include a TME rich in inhibitory prostaglandins (e.g. PGE2) or adenosine and one where electrolyte imbalances many impact upon transmembrane ion shifts vital for T-cell effector function. Recombinant OVs may be able to reverse this immunosuppressive TME by targeting specific metabolic pathways inhibitory to T-cell effector function. Many of these strategies have not yet been investigated in experimental paradigms with CAR T-cell therapy.

The COX2 / PGE2 pathway has been associated with attracting and maintaining the suppressive phenotype of MDSCs within the TME [175]. PGE2 is increasingly recognised as a key mediator of resistance to a variety of immunotherapies reliant upon T-cell function, including immune checkpoint blockade [176]. In addition to the aforementioned oncolytic vaccinia OV expressing HPGD (WR.TK-HPGD+), an oncolytic HSV-1 armed with HPGD was found to mitigate immunosuppression mediated by PGE2 and exhibited anti-tumour effects in an ectopic primary and metastatic breast cancer model in mice [177].

Indoleamine 2,3-dioxygenase 1/2 (IDO1/2) and the closely related tryptophan 2,3-dioxygenase (TDO) represent the first enzymatic step in the catalysis of the amino acid L-tryptophan into N-formyl-L-kynurenine [178]. The dual impact of enhanced IDO/TDO activity on decreasing tryptophan and increasing kynurenine leads to a number of immunosuppressive effects, including reduced T-cell activation and proliferation [179], reduced TCR signalling [180] and enhanced Treg function [181]. Whilst there are no reported recombinant OVs armed with IDO pathway inhibitors, in human glioma cells infected with *either* a wild-type oncolytic HSV strain (KOS) *or* one edited to remove a viral virulence gene (ICP0) designed to counteract the normal IFNγ response (JD05), IFNγ-induced IDO production was found to be almost completely abrogated [182]. Vector-induced IDO downregulation may have potential for synergistic combinatorial use with a whole host of immunotherapeutic modalities including CAR T-cell therapy.

Adenosine is synthesized from adenosine monophosphate (AMP) by the ectoenzyme CD73 and exhibits pluripotent immunosuppressive effects in the TME, including the impairment of NK and CD8^+^ T-cell cytotoxic effector function and a polarizing effect on myeloid cells to differentiate into immunosuppressive phenotypes such as M2 macrophages and DCs [183]. Small molecule inhibitors of CD73 and A2AR are in clinical development [184] but thus far, no published data describes a recombinant OV designed to circumvent this pathway. A recently reported study has demonstrated a profound increase in CAR T-cell efficacy following genetic (shRNA) or pharmacological inhibition of A2AR, particularly in combination with PD-1 blockade [185]. Furthermore, CAR T-cells specifically engineered to mitigate the immunosuppressive effect of enhanced adenosine levels in the TME shall be described later in this review.

Hypoxia has been found to reduce the replication of OVs in cancer cells by mediating a downregulation of viral protein expression [186]. However, this is not universal. Newcastle disease virus, for example, exhibits increased oncolytic activity in hypoxic cancer cells and can diminish hypoxia-induced HIF-1α accumulation, a pro-survival pathway that is upregulated in a number of tumours [187]. As such, it may represent a good partner for cellular therapies targeting hypoxic solid tumours. The lower pH found in many tumours has also been addressed by the development of a pH-sensitive and bio-reducible polymer (PPCBA)-coated oncolytic adenovirus (Ad-PPCBA). This adenoviral derivative was able to not only infect cancer cells at the low pH found in hypoxic tumours but was able to circumvent the requirement for viral entry via the coxsackie and adenovirus receptor (CAR) and infect cells via micropinocytosis [188].

### Delivery of pro-apoptotic or cytotoxic molecules

A large number of “suicide” transgenes have been explored in recombinant OVs, particularly at a time when researchers placed greater import on oncolytic potential rather than immunogenicity. Whilst the latter has now emerged at the forefront of OV development, it remains linked to OV-induced cell death triggering the release of neo-antigens that can engender long-lasting adaptive immune responses. Oncolysis is in itself capable of causing tumour regression in vivo and its importance in immune competent hosts is likely to be enhanced by the optimisation of OV treatment schedules as well as techniques to limit OV neutralisation by the host immune system. Due to the complex interplay between OV infection and immune clearance it is likely that the insertion of gene switches providing regulatory temporal control of oncolysis may prove particularly efficacious.

Enhanced oncolytic potential is therefore be expected to have broad synergism with CAR T-cell mediated cytotoxicity. Novel OV constructs have incorporated numerous pro-apoptotic molecules including TRAIL [189], TNFα [190], apoptin [26] and artificial death receptor ligand fusion molecules (e.g. FasL-HER2) [191]. Other suicide transgenes have encoded bacterial cytosine deaminase (CD) which is able to convert 5-fluorocytosine into the cytotoxic molecule 5-fluorouracil (5-FU) [192]; and adenovirus death protein (ADP), a nuclear membrane glycoprotein required for efficient adenoviral cell lysis, which can induce enhanced oncolysis if overexpressed following insertion into the adenoviral E3 locus [193].

Spatial control over apoptotic signalling may be achieved using host tissue-enriched or tissue-specific promoters capable of preferentially expressing suicide genes within cancer cells. One example is a mutant adenovirus (Ad-OC-HSV-TK) designed to target bone tumours. In this construct, the HSV-1 TK gene is driven by the osteocalcin promoter. Following exposure to thymidine analogues (such as ganciclovir), osteosarcoma cells infected by the OV undergo DNA synthesis arrest leading to cell death [194].

Cationic antimicrobial peptides (CMPs), small molecules capable of disrupting lipid membranes due to cationic and amphipathic properties, have been utilised in recombinant OVs due to their tumouricidal and immune modulatory effects. Bovine lactoferricin, a CMP isolated from cow’s milk following acid-pepsin hydrolysis of lactoferrin, has demonstrated potent cytotoxic effects against a number of murine and human cancer cell lines in vitro and in vivo [195]. A chemically modified peptide form of bovine lactoferricin known as LTX-315 was shown to induce the release of DAMPS associated with immunogenic cell death [196]. A recombinant Lister strain vaccinia OV armed with both lactaptin, a pro-aopototic proteolytic fragment of kappa-casein found in human breast milk, and GM-CSF was found to demonstrate enhanced tumouricidal function in vitro compared to a GM-CSF-armed control. Furthermore, the dual armed OV was able to induce significantly delayed tumour growth in both a chemosensitive and chemoresistant mouse tumour model [197].

Oncolytic viruses have also been combined with other pro-apoptotic molecules, and these synergistic combinations may be further exploited by combination with cellular therapies such as CAR T-cell therapy. In particular, second mitochondria-derived activator of caspases (Smac) mimetic compounds (SMCs), which, by countering inhibitor of apoptosis proteins (IAP) are able to sensitise tumour cells to apoptotic signalling induced by inflammatory cytokines, have been used in combination with OVs to induce potent bystander tumour cell death [198]. This strategy may be particularly effective in “non-inflamed” tumours and may be further enhanced by the arming of OVs with pro-apoptotic cytokines such as type I IFNs, TNFα or TRAIL.

### Delivery of molecules capable of structurally altering the tumour microenvironment

Oncolytic virus infection may induce structural changes within the TME conducive to CAR T-cell entry and mobilisation. The dense fibrotic stroma present in a number of solid tumours enriched with MDSCs and fibroblasts constitutes a formidable physical barrier to both OV infection and T-cell entry [57]. Outside of the OV and ACT field, enzymes such as heparanase and hyaluronidase which target the fibroblast-derived extracellular matrix have been investigated in early phase clinical trials. The parenteral administration of a pegylated recombinant human hyaluronidase (pegPH20) can dramatically alter the structure of desmoplastic tumours such as PDAC. Mid-stage reporting of the phase II HALO-202 trial reports a significant progression free survival advantage (from 5.2 to 9.2 months) using pegPH20 in combination with nab-Paclitaxel and Gemcitabine in patients with advanced PDAC and high levels of hyaluronan [199]. Collagen in the ECM may be degraded by co-injection with a bacterial collagenase thus improving the spread and efficacy of an oncolytic herpes simplex virus [200]. Oncolytic viruses have therefore been engineered to express ECM-degrading enzymes such as hyaluronidase, leading to increased OV dissemination within the tumour and therapeutic activity in a melanoma xenograft model [201]. An oncolytic adenovirus expressing the collagenase matrix metalloproteinase 9 (MMP)-9 has also demonstrated improved viral spread in human pancreatic and lung cancer xenograft models [202]. Such an approach would appear to be more attractive than engineering CAR T-cells to express such enzymes given that the latter would be anticipated to encounter the same problem of TME-entry that their payload was designed to circumvent.

Oncolytic viruses may also induce direct or indirect effects on the tumour microvasculature such as enhanced vascular permeability that are expected to be synergistic with CAR T-cell therapy. Indeed, several OVs including VSV and HSV have been demonstrated to preferentially infect tumour-associated endothelial cells [203]. A number of systemically administered vaccinia strains are not only endothelial cell-tropic but harbour the capability to directly modulating the vasculature of tumours to exert an anti-tumour effect. Oncolytic viruses may be armed with anti-angiogenic molecules such as vascular endothelial growth factor (VEGF)/ VEGF receptor inhibitors (e.g. ONYX-015), anti-angiogenic cytokines e.g. IL-12, IL-18 or IL-24 or small interfering ribonucleic acids (siRNAs) targeting the pro-angiogenic cytokine IL-8. Furthermore, OVs have been armed with anti-angiogenic chemokines such as platelet factor 4 or the angiostatic C-X-C motif chemokines CXCL-10 and CXCL-12. A plethora of endogenous anti-angiogenic molecules have also been explored in recombinant OVs including endostatin, angiostatin, vasculostatin, plasminogen kringle 5, canstastin and the fibroblast growth factor receptor (FGFR) [204]. As yet, these anti-angiogenic OVs have not been explored in combination with ACT and it is unclear whether this approach may be replicated by the systemic delivery of anti-angiogenic agents already licensed for the treatment of solid tumours such as the anti-VEGFA mAb bevacizumab or the anti-VEGFR2 mAb, ramucirumab.

### Dual modification strategies involving both CAR T-cells and oncolytic viruses

A multitude of approaches designed to enhance CAR T-cell efficacy are undergoing evaluation. Enhanced tumour specificity and spatiotemporal control may be achieved with the use of co-expressed inhibitory iCARs [205] or logic-gated control circuits [206]. Inhibitory checkpoints in the TME can be harnessed to provide positive signals, for example using “switch CARs” [207] or through co-expression of secreting small molecule traps or antibodies [150]. Durability of T-cell function can be enhanced by targeting respiratory pathways within the CAR T-cells themselves. Whilst all of these approaches would all be expected to enhance CAR T-cell synergism with oncolytic virotherapy, they represent indirect means to achieve this.

One method, however, that may foster direct synergism involves chemokine receptor and ligand matching. Newick et al. engineered mesothelin-redirected CAR T-cells to express a “regulatory subunit I anchoring disruptor” (RIAD) that blocks the association of protein kinase A (PKA) with the transmembrane molecule ezrin, thus preventing adenosine or PGE2-mediated TCR inactivation. By this means, CAR T-cell trafficking and anti-tumour efficacy could be augmented. These CAR T-cells demonstrated increased TCR signalling, enhanced cytokine production and more robust effector function. However, their increased efficacy in vivo was also found to be dependent upon secondary overexpression of CXCR3, the main receptor for the IFNγ-dependent chemokine ligands CXCL-9 and CXCL-10 found at elevated levels the TME of inflamed tumours [208]. CXCR3 upregulation occurs rapidly in T-cells following DC-induced activation and prior to proliferation in an antigen-specific manner. Since CAR T-cell function bypasses the need for DC engagement and cells are activated ex vivo, CXCR3 upregulation may be not be sustained to facilitate migration to the TME. The forced upregulation of CXCR3 is therefore particularly pertinent to CAR T-cell therapy over other forms of ACT. The approach is also attractive as it may allow anti-tumour responses to access a CXCR3-chemokine dependent amplification loop whereby local CXCR3 ligand expression in the TME (potentially induced by non-specific OV-mediated IFNγ release or by recombinant engineering) promotes the additional recruitment of CXCR3^+^ CAR T-cells, which in turn secrete IFNγ locally activating the transcription factor STAT1 in TME resident cells further amplifying the infiltration of CAR T-cells [209]. In CAR T-cell therapy, this feedback loop may provide a means of replicating the “inflamed” immunophenotype of tumours enriched in IFNγ due to DC-mediated antigen presentation to CD4^+^ and CD8^+^ T-cells.

With the aim of improving mesothelin-directed CAR (mesoCAR) T-cell therapy for malignant pleural mesothelioma (MPM), investigators sought to match-up tumour-specific chemokine secretion with the forced expression of a matching chemokine receptor. Of 20 chemokines and cytokines examined from the supernatant of 11 human MPM cell-lines, CCL2 stood out as one of the most highly and uniformly expressed. MesoCAR T-cells engineered to co-express the CCL-2 receptor CCR2b exhibited significantly enhanced intra-tumoural trafficking and anti-tumour effects [210]. Such an approach may be expected to function synergistically with an MPM-tropic recombinant OV armed with CCL2.

In a non-CAR model of ACT, enhanced chemokine ligand / receptor matching was demonstrated using adoptively transferred T-cells engineered to express CX3CR1 (the fractalkine receptor), leading to enhanced homing towards CX3CL1-producing tumours and improved efficacy in an immunodeficient mouse xenograft model [211]. These studies provide further evidence that correcting the chemokine gradient between the systemic circulation and the tumour is likely to be an essential requirement for effective ACT including CAR T-cell therapy. Suggested strategies may therefore include OV-mediated overexpression of CCL-5 matched with CAR T-cell overexpression of CCR1, CCR3 or CCR5; matched overexpression of CXCL-11 and CXCR3; or CCL-19 and CCR7; and CX3CL1 and CX3CR1. Although to a certain extent, this is provided by non-chemokine receptor-engineered CAR T-cells which retain CCR1, CCR3, and CCR5 expression following ex vivo expansion, additional modification of the CAR T-cell may facilitate more robust homing to a specific TME. Such an approach may be optimally tailored by choosing a chemokine ligand and receptor matching pair that overlaps with physiological chemokine ligand expression in specific tumours e.g. using CX3CL1/CX3CR1 matching in ovarian cancer [128] or CCL-19/CCR7 matching in breast cancer [212].

One safety concern regarding the forced upregulation of baseline chemokine receptors in ACT is the risk of inducing autoimmunity. Both CXCR3^+^ and CCR5^+^ T-cells have been found to associate with inflammatory lesions in a number of autoimmune diseases, e.g. colonic mucosa in ulcerative colitis and myelinated neural tissue in multiple sclerosis [213]. The use of CAR T-cells which can function independently of antigen cross-presentation may be far less prone to this than polyclonal TCR TIL-based therapy. Strategies designed to render TCR-signalling non-functional or delete the TCR complex itself are likely to mitigate this risk.

An alternative matching strategy may involve the matched overexpression of co-stimulatory ligands and receptors by OV-infected cancer cells and CAR T-cells respectively. For example, OVs encoding B7.1 and/or B7.2 may enhance the functionality of CAR T-cells that express endogenous CD28. Similarly, OV encoding CD40, CD40L, 4-1BBL or OX40L may be utilised synergistically with CAR T-cells that express the appropriate counter-receptor.

### Delivery of oncolytic viruses by CAR T-cells or tumour-infiltrating lymphocytes

The systemic delivery of OVs has been compromised by technical and clinical challenges. These range from the induction of severe cytokine-mediated toxicity (e.g. systemic adenoviral OV administration) to impaired infectivity and persistence due to host immune evasion. A number of groups have highlighted the potential of cellular therapies (including CAR T-cells) to deliver OVs more effectively to their target in vivo [214–216]. Such an approach potentially circumvents many of the challenges with systemic OV delivery and poor tumour targeting and infectivity. The repeated administration of OVs induce antibody and complement-induced immune evasion and viral clearance. The effect is amplified in previously immunized individuals. Whilst various conditioning strategies have been employed to mitigate this effect e.g. utilising cytotoxic agents or targeted therapies such as cyclophosphamide or rituximab to deplete B-cells [31] or complement inhibition (e.g. in preclinical models using compstatin, a 13 amino acid cyclic peptide able to bind to human C3 and C3b [30] or by using sub-lethal dose cobra venom factor [217]), the carriage of OVs by cellular products such as CAR T-cells may shield them from the host immune system whilst inducing a synergistic bystander effect in the TME. However, whilst this approach appears elegant, OV-infected CAR T-cells are still expected to encounter the same barriers to T-cell entry seen in many solid tumour models. Alternative approaches designed to overcomes immune suppression include the selection of specific OV strains intrinsically resistant to antibody or complement-mediated clearance (e.g. extracellular enveloped virus (EEV)-high VVs [218]) or by introducing novel structural changes to the viral coating itself e.g. with multi-layer ionic polymers [219].

## Conclusions, outstanding questions and potential future strategies

The number of potential strategies to fruitfully combine OV and CAR T-cell immunotherapy are legion. Due to the complexity of OV and immune cell inter-relationships, optimal synergy between these two immunotherapeutic modalities is unlikely to benefit from the optimisation of each arm independently. Oncolytic virus infection, for example, may be forestalled by the injudicious adaptation of CAR T-cells able to elicit potent anti-viral effects. Likewise, CAR T-cell function is unlikely to be enhanced in a synergistic fashion by using an OV with improved oncolytic function mediated by apoptotic, rather than immunogenic cell death. Instead, a more considered systems-based approach is likely to be more profitable when combining OVs with solid tumour-redirected CAR T-cells. Considerable work is required to counteract the immunosuppressive milieu within many solid tumours, which is both non-permissive to CAR T-cell entry and non-conducive to CAR T-cell expansion and anti-tumour effector function. It remains to be seen whether sufficiently potent and selective OVs can be designed to negate these barriers to effective CAR T-cell therapy in future clinical trials.

Given the plethora of OV families and strains currently under investigation, it is unclear which OV subtypes will prove most conducive to synergistic combination with individual CAR T-cell products. In view of the great diversity of tumour-tropism, specificity, transgene carriage capacity and impact upon host innate and adaptive immune responses it is likely that individual OVs will be selected for their particular attributes and ability to counteract the specific problems encountered by each specific CAR T-cell strategy. With the FDA’s recent approval of tisagenlecleucel for children and young adults with ALL, it is intriguing to consider whether CAR and OV synergism could be evaluated in haematological malignancy, thus potentially accelerating proof of principle. Certainly, a number of OVs (such as parvovirus and NDV) have been evaluated in preclinical models of leukaemia, lymphoma and myeloma [220, 221]. The local induction of innate immunity following OV infection coupled with the potential to enhance CAR T-cell homing and effector function by inserting specific transgenes into the OV genome may be expected to yield improved results with CD19-directed CAR T-cells. However, the considerable single agent efficacy of the latter in this setting would suggest that the bar to demonstrating effective synergism may be significantly higher than in solid tumours.

Several questions remain regarding the optimal combination of OVs and CAR T-cell ACT. Will optimal administration of OVs (and indeed CAR T-cells) be achieved by local intra-tumoural injection or by systemic parenteral delivery? Which CAR intracellular domain/construct may be most conducive to synergy with specific OVs? Which tumours are likely to be optimal targets for synergistic combination? Will the optimal combined use of (engineered) OV differ in the treatment of “hot” (inflamed) as opposed to “cold” non-inflamed tumours? Other questions relate to the optimal disease setting within which such experimental approaches could eventually be deployed. Whilst advanced cancer has proven to be more resistant, early stage disease (such as localised breast cancer, which is commonly cured with a combination of surgery, radiotherapy and hormone therapy) remains challenging to treat using cellular therapies, due to the requirement for highly toxic lymphodepleting conditioning (often incorporating cyclophosphamide with fludarabine) with or without the administration of systemic cytokines such as IL-2. There is indirect evidence, however, that OV infection may obviate the need for such pre-conditioning and intra-tumoural delivery of OVs and CAR T-cells would be expected to permit the treatment of early stage disease in a more controlled and less toxic manner. Alternatively, there may be a particular role for the synergistic combination of OVs and CAR T-cell therapy in patients who have progressed following immune checkpoint blockade. Review of patients who have responded to anti-CTLA-4 and/or anti-PD-1/PD-L1 axis blockade over many years has yielded valuable data about the mechanisms of acquired resistance [42]. As a final consideration, OV infection may prove to be more efficacious in tumours with an acquired impairment of IFNγ signalling (e.g. through mutation of the JAK-STAT pathway), whereas CAR T-cell therapy may provide a more attractive option for patients whose tumours have lost MHC class I expression (e.g. due to β2-microglobulin mutations). Certainly, the rapid technical advancement and falling cost of developing genetically engineered biotherapies heralds the imminent arrival of individualised cellular and viral products for the treatment of cancer.

## Conclusions

The potential for combining OVs, CAR T-cell therapy and an additional immunotherapeutic strategy are practically limitless. Furthermore, due to the broad applicability of these techniques the ability to synergistically combine OVs with TCR-engineered ACT (e.g. targeting cancer testis antigens such as NY-ESO-1) may also be considered. Triplet or quadruplet combinations may be envisaged using systemically delivered immune checkpoint inhibitors and/or co-stimulatory agonists, or indeed other immunomodulatory agents such as IDO inhibitors, TLR or STING agonists, Smac mimetics, A2R antagonists, anti-angiogenic agents or even novel DGK and SHP-1 inhibitors. The timing and scheduling of these combination strategies is likely to be crucial. For example, studies have shown that administering an anti-CTLA-4 mAb on the same day as delivering a vaccinia OV can impair efficacy of the latter, presumably due to an enhanced anti-viral effect. Such combinations are also likely to be dependent upon the family and strain of the OV used, and indeed the synergistic combination of an anti-CTLA-4 mAb was found to be strain specific [222]. In short, the future is likely to yield a rich seam of translational and clinical research in which these two exciting technologies are combined to tackle the pressing need for improved treatments for patients with advanced solid tumours.

## Abbreviations

5-FU: 5-fluorouracil
A2AR: Adenosine A2a receptor
ACT: Adoptive cell transfer
ADP: Adenovirus death protein
ALL: Acute lymphoblastic leukaemia
AMP: Adenosine monophosphate
ATP: Adenosine triphosphate
ATRA: All-trans retinoic acid
BCL: B-cell lymphoma
BiTE: Bispecific T-cell engager
CAF: Cancer-associated fibroblast
CAR: Chimeric antigen receptor
CCL: C-C Motif Chemokine Ligand
CCR: Co-stimulatory receptor
CD: Cytosine deaminase
CD40L: CD40 ligand
CIK: Cytokine induced killer
CLL: Chronic lymphocytic leukaemia
CMP: Cationic antimicrobial peptide
CMS: Consensus molecular subtype
COX: Cyclooxygenase
CRS: Cytokine release syndrome
CTLA-4: Cytotoxic T lymphocyte antigen 4
CXCL: C-X-C motif chemokine ligand
DAMPS: Danger associated molecular pattern signals
DC: Dendritic cell
DGK: Diacylglycerol kinase
EBV: Epstein Barr virus
EEV: Extracellular enveloped virus
EGFR: Epidermal growth factor receptor
EMT: Epithelial-mesenchymal transition
FGF: Fibroblast growth factor
FGFR: Fibroblast growth factor receptor
GITR: Glucocorticoid-induced TNFR-related protein
GM-CSF: Granulocyte macrophage colony-stimulating factor
HLA: Human leukocyte antigen
HMGB1: High-mobility group box 1
HN: Hemagglutinin-neuraminidase
HPGD: Prostaglandin dehydrogenase
HSV-1: Human herpes simplex type I virus
ICAM-1: Intercellular adhesion molecule 1
IDO: Indoleamine 2,3-dioxygenase
IFN: Interferon
IL: Interleukin
LFA: Lymphocyte function-associated antigen
mAb: monoclonal antibody
MAGE-A: Melanoma-associated antigen
MDSC: Myeloid-derived suppressor cell
MHC: Major histocompatibility complex
MMP: Matrix metalloproteinase
MPM: Malignant pleural mesothelioma
NCI: National Cancer Institute
NDV: Newcastle disease virus
NK: Natural killer
NPC: Nasopharyngeal carcinoma
NSG: NOD SCID γcnull
NY-ESO: New York oesophageal squamous cell carcinoma
OV: Oncolytic virus
PAMPS: Pathogen associated molecular pattern signals
PBMC: Peripheral blood mononuclear cell
PCR: Polymerase chain reaction
PD-1: Programmed cell death protein 1
PDAC: Pancreatic ductal adenocarcinoma
pegPH20: pegylated recombinant human hyaluronidase
PGE2: Prostaglandin E2
PKA: Protein kinase A
PKR: Protein kinase R
RAGE: Receptor for Advanced Glycation End Products
RANTES: Regulated on activation, normal T cell expressed and secreted
RIAD: Regulatory subunit I anchoring disruptor
scFv: single chain variable fragment
shRNA: short hairpin RNA
siRNA: small interfering ribonucleic acid
SMC: Smac mimetic compound
STING: Stimulator of Interferon Genes
TAA: Tumour-associated cell surface antigen
TAM: Tumour-associated macrophages
TAP: Transporter associated with antigen processing and presentation
TBK: TANK-binding kinase
TCR: T-cell receptor
TDO: Tryptophan 2,3-dioxygenase
TGF-β: Transforming growth factor beta
TIL: Tumour infiltrating lymphocyte
TK: Thymidine kinase
TLR: Toll-like receptor
TME: Tumour microenvironment
TNFα: Tumour necrosis factor-alpha
TRAF: TNF receptor-associated factor
TRAIL: TNF-related apoptosis-inducing ligand
Treg: Regulatory T-cell
TSA: Tumour-specific cell surface antigen
T-VEC: Talimogene laherparepvec
VEGF: Vascular endothelial growth factor
VSV: Vesicular stomatitis virus
VV: Vaccinia virus
vvDD: Double-deleted vaccinia virus
WR: Western Reserve
